# Challenging inflammatory process at molecular, cellular and in vivo levels via some new pyrazolyl thiazolones

**DOI:** 10.1080/14756366.2021.1887169

**Published:** 2021-02-22

**Authors:** Perihan A. Elzahhar, Rana A. Alaaeddine, Rasha Nassra, Azza Ismail, Hala F. Labib, Mohamed G. Temraz, Ahmed S. F. Belal, Ahmed F. El-Yazbi

**Affiliations:** a Department of Pharmaceutical Chemistry, Faculty of Pharmacy, Alexandria University, Alexandria, Egypt; b Department of Pharmacology and Toxicology, Faculty of Medicine and Medical Centre, American University of Beirut, Beirut, Lebanon; c Department of Medical Biochemistry, Faculty of Medicine, Alexandria University, Alexandria, Egypt; d Department of Pharmaceutical Chemistry, College of Pharmacy, Arab Academy of Science Technology and Maritime Transport, Alexandria, Egypt; e Faculty of Pharmacy, Alexandria University, Alexandria, Egypt; f Department of Pharmacology and Toxicology, Faculty of Pharmacy, Alexandria University, Alexandria, Egypt

**Keywords:** Pyrazolyl thiazolones, anti-inflammatory, cyclooxygenase-1/cyclooxygenase-2, 15-lipoxygenase, macrophage apoptosis

## Abstract

The work reported herein describes the synthesis of a new series of anti-inflammatory pyrazolyl thiazolones. In addition to COX-2/15-LOX inhibition, these hybrids exerted their anti-inflammatory actions through novel mechanisms. The most active compounds possessed COX-2 inhibitory activities comparable to celecoxib (IC_50_ values of 0.09–0.14 µM) with significant 15-LOX inhibitory activities (IC_50_s 1.96 to 3.52 µM). Upon investigation of their *in vivo* anti-inflammatory activities and ulcerogenic profiles, these compounds showed activity patterns equivalent or more superior to diclofenac and/or celecoxib. Intriguingly, the most active compounds were more effective than diclofenac in suppressing monocyte-to-macrophage differentiation and inflammatory cytokine production by activated macrophages, as well as their ability to induce macrophage apoptosis. The latter finding potentially adds a new dimension to the previously reported anti-inflammatory mechanisms of similar compounds. These compounds were effectively docked into COX-2 and 15-LOX active sites. Also, *in silico* predictions confirmed the appropriateness of these compounds as drug-like candidates.

## 1. Introduction

In the last two decades, progressive steps were made in our understanding of the molecular mechanisms of Arachidonic acid (AA)-mediated inflammation^1^. Two main pathways involving either cyclooxygenase (COX) or lipoxygenase (LOX) enzymes exist with potential pro-inflammatory products. COX isoforms are responsible for the conversion of AA to prostaglandins (PGs), prostacyclin (PGI2) and thromboxane A2 (TXA2)^2–4^. While COX-1 is constitutively active and synthesises PGs with a favourable physiological role in the gastrointestinal tract and kidneys^2–4^, COX-2 expression is triggered by pro-inflammatory stimuli and is responsible for the production of PGs involved in inflammation^2–5^. On the other hand, LOX converts AA to leukotrienes (LTs) and/or eoxins^6–9^. Specifically, the latter are products of the 15-LOX pathway^10^ and together with PGs and LTs, have been implicated in the pathophysiology of several inflammatory disorders, like Alzheimer’s disease, rheumatoid arthritis, osteoarthritis, COPD, psoriasis and multiple sclerosis^6–9^. Furthermore, the perspective of developing selective COX-2 inhibitors that are devoid of GIT side effects turned out to be counterintuitive, since clinical practice proved that several of these selective inhibitors were associated with severe cardiovascular complications^11^^,^^12^. Also, only blocking the inflammatory pathway downstream of COX-2 would divert the arachidonic acid inflammatory flux into the LOX pathway; increasing the level of production of LTs/eoxins, therefore resulting in a greater incidence of unfavourable side effects such as asthma^13^. As such, constructing a dual COX-2/LOX inhibitor seems to be an advantageous therapeutic option in terms of both efficacy and safety.

**Figure 1.**
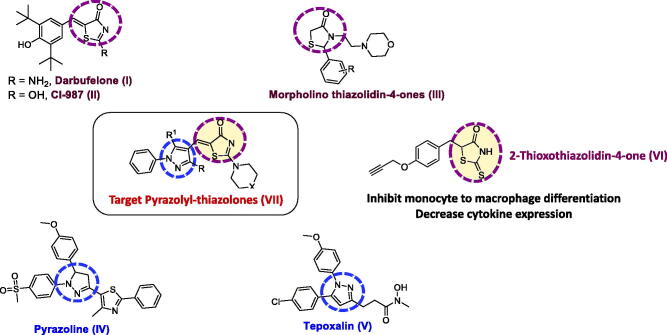
Rationale for the design of target compounds.

Up until the moment, the only *bona fide* selective COX-2/LOX inhibitor close to therapeutic use is licofelone, which entered phase 3 clinical trials for osteoarthritis^14^. Nonetheless, numerous research groups synthesised dual COX/LOX inhibitors. Of particular interest to this study, are the phenolic arylidene thiazolidinones darbufelone (Structure I, Figure 1)^15^ and CI-987 (Structure II, Figure 1)^16^ that showed low ulcerogenicity and potent COX/LOX inhibitory activity. Moreover, some morpholinoethyl thiazolidin-4-ones (Structure III, Figure 1) reduced ear oedema in mice and possessed COX inhibitory activity^17^. Besides, the pyrazoline derivative (Structure IV, Figure 1) reported by Abdellal *et al*. showed more selectivity than celecoxib towards COX-2 and more potency than meclofenamate sodium towards 15-LOX^18^. Furthermore, pyrazole-containing hydroxamic acid COX/LOX inhibitor, tepoxalin (Structure V, Figure 1), was authorised for use in veterinary settings in the European Union and the United States under the brand name Zubrin^®^ for the relief of pain associated with musculoskeletal disorders^19^.

On the other hand, monocyte recruitment and subsequent activation into macrophages is an early event of the inflammatory response^20^. This process has been recognised to play an essential role in the pathogenesis of atherosclerosis^21^. Multiple studies have reported an enhancement in COX-2 expression during monocyte differentiation into macrophages^22^^,^^23^. Prostaglandin E2, upon binding to EP2 and EP4 receptors, was shown to stimulate interleukin-10 (IL-10) production, which mediates functional reprogramming of monocytes and macrophages^24^. Additionally, 15-LOX was shown to contribute to macrophage activation and adhesion^25^. Of particular interest, 15-LOX was found to be upregulated in macrophage-rich atherosclerotic lesions^26^^,^^27^. Our previous reports demonstrated inhibitory effects of combined COX-2 and 15-LOX blockade on THP-1 monocyte-to-macrophage differentiation as shown by the 2-thioxothiazolidin-4-one derivative (Structure VI, Figure 1)^28^^,^^29^.

In view of the above facts, we envisioned that a hybrid design that combines both pyrazolyl and thiazolyl privileged scaffolds might be a good candidate for a dual COX-2/LOX inhibitor. In addition, we reasoned that adding a cyclized secondary amine moiety (such as; morpholine, piperidine and piperazine) might enhance the anti-inflammatory activity as indicated by several reports^17^^,^^30^^,^^31^. Consequently, we synthesised and evaluated the anti-inflammatory activity of the designed pyrazolyl-thiazolones (Structure VII, Figure 1). *In vitro* COX-1/2 and 15-LOX inhibitory assays were performed. Moreover, anti-inflammatory activities were assessed in several *in vivo* and *in vitro* models. Finally, docking and drug likeness studies were carried out to further support their mechanism of action and appropriateness as drug-like candidates.

## 2. Experimental

### 2.1. Chemistry

All chemicals and solvents were obtained from Sigma-Aldrich or Fisher Scientific. A Stuart melting point apparatus (SMP10) was used for determining uncorrected melting points. Infra-red spectra (IR) were recorded using KBr discs on a Shimadzu IR 435 spectrophotometer. Nuclear magnetic resonance (^1^H NMR and ^13 ^C NMR) spectra were recorded on a Bruker spectrometer (400 MHz) using deuterated Pyridine (Pyridine-*d*5) as a solvent. Elemental analyses (C, H, N and S) were conducted on a FLASH 2000 CHNS/O Analyser (Thermo Scientific). In addition, compounds were found to be ≥95% pure by reversed phase HPLC analysis using Agilent 1260 infinity HPLC equipped with G1311B Quaternary pump, G1329 injector and G 1315 D DAD Vl detector. A G1316A C18 column (4.6 × 150 mm) was used. An injection volume of 0.5 ml (DMF and phosphate buffer pH 5 1:1), a flow rate of 1 ml/min and an isocratic elution of acetonitrile in water (1:1) were applied. The detection was done at a wavelength of 254 nm.

#### 2.1.1. General procedure for the preparation of (Z)-5-benzylidene thiazol-4(5H)-one (1-10):

A mixture of the appropriate aldehyde a-e (1.5 mmol), rhodanine (0.13 g, 1 mmol) and cyclic secondary amine (1.5 mmol) in absolute ethanol (10 ml), in the presence of catalytic amount of glacial acetic acid was heated under reflux with stirring for 12–15 h. After cooling to room temperature overnight, the precipitated product was filtered, washed with cold ethanol and then dried. The solid was crystallised from ethanol or ethanol/DMF to furnish the appropriate solid products.

##### 2.1.1.1. (Z)-5-((5-chloro-3-methyl-1-phenyl-1H-pyrazol-4-yl)methylene)-2-(piperidin-1-yl)thiazol-4(5H)-one (1)

Yield 84.9%. m.p0.184–186 °C. IR (KBr, cm^−1^): 1589.34 (C = N), 1678.07 (C = O), 2924.09 (aliphatic C-H). ^1^H NMR (400 MHz, Pyridine-*d*_5_): *δ* 1.37–1.42 (*m*, 6H, piperidine-C_3,4,5_-H), 2.37 (*s*, 3H, CH_3_), 3.33 − 3.35 and 3.87 (2* m*, 4H, piperidine-C_2,6_-H), 7.35-7-39 (*t*, *J* = 8 Hz, 1H, Aryl-C_4_-H), 7.46–7.50 (*t*, *J* = 8 Hz, 2H, Aryl-C_3,5_-H), 7.70–7.72 (d, *J* = 8 Hz, 2H, Aryl-C_2,6_-H), 7.87 (*s*, 1H, =CH). ^13 ^C NMR (100 MHz, Pyridine- *d*_5_): *δ* 179.7, 173.8, 148.9, 138.2, 132.5, 129.3, 128.7, 128.6, 126.1, 125.05, 119.4, 114.8, 49.8, 49.2, 26.0, 25.3, 23.8, 13.8. Anal. Calcd (%) for C_19_H_19_ClN_4_OS (386.90): C, 58.98; H, 4.95; N, 14.48; S, 8.29. Found: C, 59.12; H, 4.98; N, 14.67; S, 8.38. HPLC/DAD: Retention time 6.49 min and 100% purity.

##### 2.1.1.2. (Z)-5-((5-chloro-3-methyl-1-phenyl-1H-pyrazol-4-yl)methylene)-2-morpholinothiazol-4(5H)-one (2)

Yield 91.1%. m.p 0.215–217 °C. IR (KBr, cm^−1^): 1616.35 (C = N), 1678.07 (C = O), 2974.23 (aliphatic C-H). ^1^H NMR (400 MHz, Pyridine-*d*_5_): *δ* 2.38 (*s*, 3H, CH_3_), 3.52 − 3.54 and 3.66–3.69 (2 *m*, 4H, morpholine-C_2,6_-H), 3.71 − 3.74 and 4.00–4.02 (2 *m*, 4H, morpholine-C_3,5_-H), 7.36–7.40 (dd, *J* = 7.2 & 7.6 Hz, 1H, Aryl-C_4_-H), 7.47-7.51 (dd, *J* = 7.6 & 8 Hz, 2H, Aryl-C_3,5_-H), 7.70–7.72 (d, *J* = 8 Hz, 2H, Aryl-C_2,6_-H), 7.90 (*s*, 1H, =CH). ^13 ^C NMR (100 MHz, Pyridine- *d*_5_): *δ* 179.5, 174.9, 148.9, 138.1, 131.6, 129.3, 129.3, 129.1, 128.6, 126.2, 125.0, 120.1, 114.6, 66.1, 66.0, 48.6, 48.5, 13.8. Anal. Calcd (%) for C_18_H_17_ClN_4_O_2_S (388.87): C, 55.60; H, 4.41; N, 14.41; S, 8.24. Found: C, 55.84; H, 4.38; N, 14.63; S, 8.30. HPLC/DAD: Retention time 4.92 min and 99.79% purity.

##### 2.1.1.3. (Z)-5-((1,3-diphenyl-1H-pyrazol-4-yl)methylene)-2-(piperidin-1-yl)thiazol-4(5H)-one (3)

Yield 92.7%. m.p 0.260–262 °C (reported 262–264 °C^32^). IR (KBr, cm^−1^): 1573.91 (C = N), 1674.21 (C = O), 2927.94 (aliphatic C-H). ^1^H NMR (400 MHz, Pyridine-*d_5_*): *δ* 1.426 (*m*, 6H, piperidine-C_3,4,5_-H), 3.19–3.30 & 3.87 (2 *m*, 4H, piperidine-C_2,6_-H), 7.32–8.12 (*m*, 10H, Ar-H), 8.19 (*s*, 1H, =CH), 8.71 (*s*, 1H, pyrazole-C_5_-H). ^13 ^C NMR (100 MHz, Pyridine-*d_5_*): *δ* 180.2, 173.2, 154.0, 139.7, 135.9, 134.8, 132.6, 129.7, 129.4, 129.1, 129.0, 128.9, 127.4, 127.2, 123.8, 122.8, 120.2, 119.4, 117.6, 49.8, 49.2, 26.0, 25.3, 23.8. Anal. Calcd (%) for C_24_H_22_N_4_OS (414.53): C, 69.54; H, 5.35; N, 13.52; S, 7.73. Found: C, 69.80; H, 5.48; N, 13.74; S, 7.65. HPLC/DAD: Retention time 11.25 min and 98.60% purity.

##### 2.1.1.4. (Z)-5-((1,3-diphenyl-1H-pyrazol-4-yl)methylene)-2-morpholinothiazol-4(5H)-one (4)

Yield 86.4%. m.p 0.263–265 °C (reported 266–268 °C^32^). IR (KBr, cm^−1^): 1573.91 (C = N), 1678.07 (C = O), 2974.23 (aliphatic C-H). ^1^H NMR (400 MHz, Pyridine-*d*_5_): *δ* 3.47 − 3.54 and 3.67–3.69 (2 m, 4H, morpholine-C_2,6_-H), 3.71–3.74 and 4.00–4.02 (2 *m*, 4H, morpholine-C_3,5_-H), 7.34-8.11 (*m*, 10H, Ar-H), 8.19 (*s*, 1H, =CH), 8.54 (*s*, 1H, pyrazole-C_5_-H). ^13 ^C NMR (100 MHz, Pyridine-*d*_5_): *δ* 179.9, 174.2,170.6, 154.5, 139.7, 132.5, 129.7, 129.1, 128.8, 128.0, 127.6, 127.4, 126.0, 122.0, 120.9, 119.69, 119.4, 117.5, 116.6, 66.1, 66.0, 48.7, 48.5. Anal. Calcd (%) for C_23_H_20_N_4_O_2_S (416.50): C, 66.33; H, 4.84; N, 13.45; S, 7.70. Found: C, 66.54; H, 4.90; N, 13.62; S, 7.76. HPLC/DAD: Retention time 8.50 min and 100% purity.

##### 2.1.1.5. (Z)-5-((1-phenyl-3-(p-tolyl)-1H-pyrazol-4-yl)methylene)-2-(piperidin-1-yl)thiazol-4(5H)-one (5)

Yield 84.6%. m.p 0.203–205 °C. IR (KBr, cm^−1^): 1597.06 (C = N), 1678.07 (C = O), 2931.80 (aliphatic C-H). ^1^H NMR (400 MHz, Pyridine-*d_5_*): *δ* 1.45 (*m*, 6H, piperidine-C_3,4,5_-H), 2.29 (*s*, 3H, CH_3_), 3.34 & 3.90 (2 *m*, 4H, piperidine-C_2,6_-H), 7.28–7.93 (*m*, 9H, Ar-H), 8.13 (*s*, 1H, =CH), 8.57 (*s*, 1H, pyrazole-C_5_-H). ^13 ^C NMR (100 MHz, Pyridine-*d_5_*): *δ* 180.31, 173.2, 170.6, 154.7, 139.0, 138.7, 129.8, 129.1, 128.0, 127.6,127.2, 125.8, 122.8, 122.2, 120.4, 119.6, 119.4, 117.6, 116.6, 49.9, 49.2, 26.0, 25.3, 23.8, 21.0. Anal. Calcd (%) for C_25_H_24_N_4_OS (428.55): C, 70.07; H, 5.65; N, 13.07; S, 7.48. Found: C, 70.31; H, 5.70; N, 13.24; S, 7.54. HPLC/DAD: Retention time 12.62 min and 99.34% purity.

##### 2.1.1.6. (Z)-2-morpholino-5-((1-phenyl-3-(p-tolyl)-1H-pyrazol-4-yl)methylene)thiazol-4(5H)-one (6)

Yield 90.8%. m.p 0.234–236 °C. IR (KBr, cm^−1^): 1597.06 (C = N), 1678.07 (C = O), 2981.95 (aliphatic C-H). ^1^H NMR (400 MHz, Pyridine-*d*_5_): *δ* 2.26 (*s*, 3H, CH_3_), 3.45 − 3.47 and 3.66–3.68 (2 m, 4H, morpholine-C_2,6_-H), 3.70 − 3.72 and 3.99–4.01 (2* m*, 4H, morpholine-C_3,5_-H), 7.24–7.26 (d, 2H, *J* = 8 Hz, phenyl-C_2,6_-H), 7.29–7.33 (dd, *J* = 4 & 8 Hz, 1H, phenyl-C_4_-H), 7.44–7.48 (*t*, *J* = 8 Hz, 2H, phenyl-C_3,5_-H), 7.82-7.84 (d, *J* = 8 Hz, 2H, *p*-tolyl-C_3,5_-H), 8.08–8.10 (d, *J* = 8 Hz, 2H, *p*-tolyl-C_2,6_-H), 8.21 (*s*, 1H,=CH), 8.66 (s, 1H, pyrazole-C_5_-H). ^13 ^C NMR (100 MHz, Pyridine-*d*_5_): *δ* 179.9, 174.2, 154.2, 139.7, 138.7, 130.1, 129.7, 129.6, 129.2, 129.0, 128.6, 127.7, 127.3, 127.2, 121.0, 119.5, 119.4, 117.4, 66.1, 66.0, 48.6, 48.5, 21.0. Anal. Calcd (%) for C_23_H_20_N_4_O_2_S (416.50): C, 66.96; H, 5.15; N, 13.01; S, 7.45. Found: C, 66.74; H, 5.19; N, 13.28; S, 7.51. HPLC/DAD: Retention time 8.70 min and 99.80% purity.

##### 2.1.1.7. (Z)-5-((3-(4-methoxyphenyl)-1-phenyl-1H-pyrazol-4-yl)methylene)-2-(piperidin-1-yl)thiazol-4(5H)-one (7)

Yield 89.1%. m.p 0.208–210 °C. IR (KBr, cm^−1^): 1612.49 (C = N), 1678.07 (C = O), 2939.52 (aliphatic C-H). ^1^H NMR (400 MHz, Pyridine-*d_5_*): *δ* 1.41 (*m*, 6H, piperidine-C_3,4,5_-H), 3.69 (*s*, 3H, OCH_3_), 3.29 & 3.86 (2 *m*, 4H, piperidine-C_2,6_-H), 7.07–7.09 (d, 2H, *J* = 8 Hz, phenyl-C_2,6_-H), 7.28-7.31 (dd, *J* = 4 & 8 Hz, 1H, phenyl-C_4_-H), 7.43–7.47 (*t*, *J* = 8 Hz, 2H, phenyl-C_3,5_-H), 7.88–7.90 (d, *J* = 8 Hz, 2H, *p*-OCH_3_-phenyl-C_3,5_-H), 8.09-8.11 (d, *J* = 8 Hz, 2H, *p*- OCH_3_-phenyl-C_2,6_-H), 8.21 (*s*, 1H,=CH), 8.53 (*s*, 1H, pyrazole-C_5_-H). ^13 ^C NMR (100 MHz, Pyridine-*d*_5_): *δ* 180.2, 173.2, 160.4, 153.9, 139.4, 139.8, 130.5, 130.4, 129.1, 127.7, 127.5, 127.2, 127.1, 124.9, 120.4, 119.5, 119.4, 117.5, 114.5, 55.1, 49.8, 49.2, 26.0, 25.3, 23.8. Anal. Calcd (%) for C_25_H_24_N_4_O_2_S (444.55): C, 67.55; H, 5.44; N, 12.60; S, 7.21. Found: C, 67.81; H, 5.52; N, 12.89; S, 7.28. HPLC/DAD: Retention time 4.57 min and 96.32% purity.

##### 2.1.1.8. (Z)-5-((3-(4-methoxyphenyl)-1-phenyl-1H-pyrazol-4-yl)methylene)-2-morpholinothiazol-4(5H)-one (8)

Yield 90.9%. m.p 0.235–237 °C. IR (KBr, cm^−1^): 1597.06 (C = N), 1678.07 (C = O), 2939.52 (aliphatic C-H). ^1^H NMR (400 MHz, Pyridine-*d*_5_): *δ* 3.47 − 3.48 and 3.68–3.69 (2 *m*, 4H, morpholine-C_2,6_-H), 3.70 (*s*, 3H, OCH_3_), 3.71 − 3.72 and 3.99–4.01 (2 *m*, 4H, morpholine-C_3,5_-H), 7.07–7.09 (d, 2H, *J* = 8 Hz, phenyl-C_2,6_-H), 7.29–7.33 (*t*, *J* = 7.2 Hz, 1H, phenyl-C_4_-H), 7.45–7.48 (dd, *J* = 7.2 & 7.6 Hz, 2H, phenyl-C_3,5_-H), 7.88-7.90 (d, *J* = 8 Hz, 2H, *p*-OCH_3_-phenyl-C_3,5_-H), 8.08–8.10 (d, *J* = 8 Hz, 2H, *p*- OCH_3_-phenyl-C_2,6_-H), 8.21 (*s*, 1H,=CH), 8.65 (*s*, 1H, pyrazole-C_5_-H). ^13 ^C NMR (100 MHz, Pyridine-*d*_5_): *δ* 179.9, 174.2, 160.4, 154.0, 139.7, 130.4, 129.7, 128.5, 127.2, 127.1, 124.8, 121.1, 119.6, 119.4, 117.3, 114.6, 66.1, 66.0, 55.1, 48.6, 48.5. Anal. Calcd (%) for C_24_H_22_N_4_O_3_S (446.53): C, 64.56; H, 4.97; N, 12.55; S, 7.18. Found: C, 64.31; H, 5.04; N, 12.68; S, 7.22. HPLC/DAD: Retention time 5.88 min and 96.12% purity.

##### 2.1.1.9. (Z)-5-((3-(4-bromophenyl)-1-phenyl-1H-pyrazol-4-yl)methylene)-2-(piperidin-1-yl)thiazol-4(5H)-one (9)

Yield 88.7%. m.p 0.266–268 °C. IR (KBr, cm^−1^): 1589.34 (C = N), 1685.79 (C = O), 2927.94 (aliphatic C-H). ^1^H NMR (400 MHz, Pyridine-*d_5_*): *δ* 1.41 (*m*, 6H, piperidine-C_3,4,5_-H), 3.31 & 3.89 (2 *m*, 4H, piperidine-C_2,6_-H), 7.34–7.66 (*m*, 5H, phenyl-H), 7.78–7.80 (d, *J* = 8 Hz, 2H, *p*-Br-phenyl-C_3,5_-H), 8.09–8.11 (d, *J* = 8 Hz, 2H, *p*-Br-phenyl-C_2,6_-H), 8.55 (*s*, 1H,=CH), 8.67 (*s*, 1H, pyrazole-C_5_-H). ^13 ^C NMR (100 MHz, Pyridine-*d*_5_): *δ* 179.5, 173.1, 153.1, 152.7, 139.6, 132.2, 131.6, 130.8, 130.3, 129.8, 128.1, 127.7, 127.4, 126.8, 121.2, 120.0, 119.7, 117.6, 116.7, 49.9, 49.2, 26.0, 25.3, 23.8. Anal. Calcd (%) for C_24_H_21_BrN_4_OS (493.42): C, 58.42; H, 4.29; N, 11.35; S, 6.50 Found: C, 58.65; H, 4.34; N, 11.61; S, 6.62. HPLC/DAD: Retention time 11.37 min and 99.82% purity.

##### 2.1.1.10. (Z)-5-((3-(4-bromophenyl)-1-phenyl-1H-pyrazol-4-yl)methylene)-2-morpholinothiazol-4(5H)-one (10)

Yield 92.7%. m.p 0.270–272 °C. IR (KBr, cm^−1^): 1610.24 (C = N), 1678.85 (C = O), 2965.51 (aliphatic C-H). ^1^H NMR (400 MHz, Pyridine-*d*_5_): *δ* 3.47 and 3.68- (2 *m*, 4H, morpholine-C_2,6_-H), 3.71 and 4.01 (2 *m*, 4H, morpholine-C_3,5_-H), 7.33–7.65 (*m*, 5H, phenyl-H), 7.77–7.79 (d, *J* = 8 Hz, 2H, *p*-Br-phenyl-C_3,5_-H), 8.09–8.11 (d, *J* = 8 Hz, 2H, *p*-Br-phenyl-C_2,6_-H), 8.45 (*s*, 1H, =CH), 8.67 (*s*, 1H, pyrazole-C_5_-H). ^13 ^C NMR (100 MHz, DMSO-d_6_): *δ* 179.3, 174.1, 152.4, 139.6, 131.2, 130.7, 129.7, 129.2, 127.4, 123.8, 122.8, 120.4, 119.9, 66.0, 48.6, 48.5. Anal. Calcd (%) for C_23_H_19_BrN_4_O_2_S (495.40): C, 55.76; H, 3.87; N, 11.31; S, 6.47. Found: C, 55.90; H, 3.85; N, 11.47; S, 6.54. HPLC/DAD: Retention time 6.88 min and 100% purity.

### 2.2. Biological evaluation

#### 2.2.1. In vitro COX-1/2 and 15-LOX inhibition assays

The capability of the target compounds to inhibit COX-1/2 and 15-LOX enzymes was tested using colorimetric COX (ovine) (Catalog No. 560131) and lipoxygenase (Catalog No. 760700) inhibitor screening assay kits, respectively, that were supplied by Cayman chemicals, Ann Arbour, MI, USA. Both assays were carried out in compliance with the manufacturer’s instructions and in accordance to prior studies^29^^,^^33^.

#### 2.2.2. In vivo anti-inflammatory activity

Protocols including animals and their care have been carried out in accordance with the Guide for the Care and Use of Laboratory Animals issued by US National Institute of Health (NIH publication No. 83-23, revised 1996) and the ethical guidelines of Alexandria University on laboratory animals. Across all experiments, sufficient care was taken to minimise discomfort or pain for animals. Adult female Wistar rats weighing 150–250 g (acquired from the Experimental Animal Centre at Alexandria University) were utilised. All animals had access to water and food *ad libitum* and were kept in a controlled environment at 23–25 °C with a 12-h dark/light cycle. Rats were acclimatised for 7 days before the experiment. Diclofenac sodium, celecoxib (from European Egyptian Pharmaceutical industries, Alexandria, Egypt) were used as references.

#### 2.2.3. Inflammatory models

The *in vivo* anti-inflammatory activity of compounds (**2,4,7-10**) was assessed both in acute and chronic inflammatory models by using the formalin-induced paw oedema^34^^,^^35^ and cotton pellet-induced granuloma screening protocols^36^, respectively. Diclofenac sodium (5 mg/kg) and celecoxib (5 mg/kg) were used as references. Animals were randomly split into six groups, where each group consisted of six rats, and was treated with different test compounds. Sets treated with celecoxib and diclofenac sodium acted as reference and those given the vehicle only (DMSO) were used as control. The same groups of rats were utilised in inflammatory models and in ulcerogenicity experiments.

#### 2.2.4. Formalin-induced paw oedema test (acute inflammation model)

A freshly prepared 5% formalin solution (prepared from 37% formaldehyde and saline (Merck, Germany)) was used as a phlogistic agent. Test and reference compounds (5 mg/kg body weight) were dissolved in DMSO and given orally with gastric gavage once per day for seven consecutive days, whereas DMSO was administered to the control group. On the 8^th^ day, the initial paw volume was determined by a Vernier calliper. Then, a subcutaneous injection of 40 μl formalin was introduced into the right hind paw of all groups under light ether anaesthesia. The paw volume was measured 4 h after the formalin injection and the oedema volume was calculated by the difference in paw volume before and 4 h after the formalin injection. The percentage inhibition of oedema (or % protection against inflammation) was calculated for each compound as previously reported^34^^,^^35^.

#### 2.2.5. Cotton pellet-induced granuloma assay (chronic inflammation model)

The rat abdomen was prepared by shaving and swabbing with 70% ethanol then two sterilised cotton pellets (each weighing 20 ± 1 mg) were subcutaneously implanted, on both sides of the abdomen under xylazine/ketamine anaesthesia (intraperitoneally, 9 mg/kg and 50 mg/kg, respectively). Intramuscular gentamycin injection (4 mg/kg) was used for three days after the experiment to guard against post-operative infection.

Test compounds, celecoxib, diclofenac sodium or vehicle (DMSO) were administered orally as before. On the 8^th^ day after implantation, rats were subjected to light ether anaesthesia.

The pellets were excised, dried at 60^0^ C for 24 h and weighed after cooling. The mean weights of the dried cotton pellets of all groups were calculated then % granuloma inhibition of all compounds was calculated relative to control^36^.

#### 2.2.6. Gastric ulcerogenic activity

The same compounds were tested for chronic gastric ulcerative symptoms^37^^,^^38^. Rat stomach was removed on the 8^th^ day of drug administration. Stomach was opened through the greater curvature, washed and kept in saline. Gross examination was carried out for any signs of haemorrhage, hyperaemia, haemorrhagic erosion or ulcers. Moreover, histopathological inspection was conducted to verify the extent of inflammatory reaction in mucosal layers^28^^,^^38^.

#### 2.2.7. Monocyte-to-macrophage differentiation assay

Monocytes were cultured and differentiated as described previously^39^. THP-1 cells (human acute monocytic leukaemia lineage, American Type Culture Collection, Manassas, VA) were seeded at a density of 20 × 10^5^ cells/ml. A set of cells were exposed to 25 nM of phorbol myristate-acetate (PMA, Calbiochem, Darmstadt, Germany) for 24 h to drive differentiation. Another set was similarly treated with PMA followed by 100 ng/ml of lipopolysaccharide (LPS, invivogen, San Diego, CA, USA) for 72 h. After incubation, the supernatant was aspirated and the density of the adherent cells was estimated using MTS colorimetric cell viability kit (Abcam, Cambridge, UK). The inhibitory actions of different compounds on the differentiation process were evaluated by pre-incubation with different concentrations of each compound for 6 h. Cell viability after treatment was normalised to the reading after PMA exposure following a 6-h incubation with DMSO. All experiments were conducted in triplicates. A positive control for the effect of COX1/COX2 inhibition on monocyte-to-macrophage differentiation was obtained by treatment with diclofenac. IC_50_ values for each compound were determined by non-linear regression as the best fit values of the log [inhibitor] vs. response curve using GraphPad Prism software.

#### 2.2.8. Cytotoxicity assay

To confirm that the previous observations were a consequence of interference of the compounds with the differentiation process rather than a reduction in monocyte viability, THP-1 monocytes were incubated with different concentrations of the compound for 30 h. No difference in cell viability was detected. Moreover, the cytotoxic effects of the drugs on differentiated macrophages were evaluated. Following the incubation of THP-1 cells with 25 nM PMA for 24 h and 100 ng/ml LPS for 24 h, the differentiated macrophages were treated with different concentrations of the compounds for 24 h. Cell viability was assessed using MTS colorimetric cell viability kit (Abcam, Cambridge, UK).

#### 2.2.9. IL-1β and procaspase-3 protein expression by Western blotting

THP-1 monocytes were exposed to 100 μM of the test compounds, activated with 25 nM PMA for 24 h followed by 100 ng/ml LPS for 72 h. At the end of the incubation period, cells were harvested, proteins were extracted and SDS-PAGE electrophoresis, blotting, and antibody probing were conducted as described previously^40^. Primary rabbit polyclonal antibodies to IL-1β, procaspase-3, and GAPDH were obtained from Abcam (Cambridge, UK). Clarity Western ECL substrate (BioRad, Hercules, CA, USA) was used to obtain a chemiluminescence signal captured by a Chemidoc imaging system (BioRad, Hercules, CA, USA). Band density was estimated using ImageJ software (National Institutes of Health, Bethesda, MD) and normalised to the GAPDH band density as a loading control.

#### 2.2.10. Caspase-3 activity determination

THP-1 cells were activated with 25 nM PMA for 24 h, followed by 100 ng/ml LPS for 4 h. For treatment, cells were exposed to 100 μM of the test compounds. Afterwards, adherent macrophages were detached using 2.5% trypsin in PBS (abcam, Cambridge, UK). Caspase activity was assessed using the Caspase-3 Assay Kit (Abcam, Cambridge, UK). Measured activity was normalised to sample protein concentration determined by Bradford protein assay.

#### 2.2.11. Statistical analysis:

Statistical analysis was performed using one-way ANOVA followed by Tukey multiple comparisons test using GraphPad Prism Software. A *p* values < 0.05 was considered statistically significant.

### 2.3. Molecular modelling and in silico studies

#### 2.3.1. Molecular docking studies on COX-2 and 15-LOX enzymes

Molecular Operating Environment software (version MOE2016.0802) was used for the docking of compounds **4** and **7** into the x-ray crystal structures of COX- 2 (co-crystallised with SC-558, PDB ID 1CX2: https://www.rcsb.org/structure/1CX2^41^) and 15-LOX enzymes (co-crystallised with RS7, PDB ID 1LOX: https://www.rcsb.org/structure/1LOX^42^), which were obtained from Protein Data Bank. The database of the test compounds was constructed by 3 D protonation, energy minimisation and partial charges calculation. As for the proteins, they were prepared by skipping the repeating chains, surfactants and water molecules. In addition, hydrogens were added, and partial charges were calculated. Compounds were then docked into the active site through the MOE-Dock panel under default settings, applying the triangle matcher as the placement method and the London dG as the primary scoring function. Further refinement using rigid receptor and GBVI/WSA dG scoring function was carried out. The output database comprised the binding energy scores in Kcal/mol. A number of conformers for each compound was generated. The pose showing the most favourable ligand-enzyme interaction together with high score was set as default.

#### 2.3.2. In silico estimation of physicochemical properties, drug-likeness and pharmacokinetics:

The pharmacokinetic profile, drug-likeness and conformity to Lipinski’s rule and physicochemical properties for the most active six compounds were assessed by Molinspiration^43^, Pre-ADMET^44^, ProTox-II^45^ (http://tox.charite.de/protox_II/) and Data warrior^46^ software, as previously reported.

## 3. Results and discussion

### 3.1. Chemistry

**Scheme 1.**
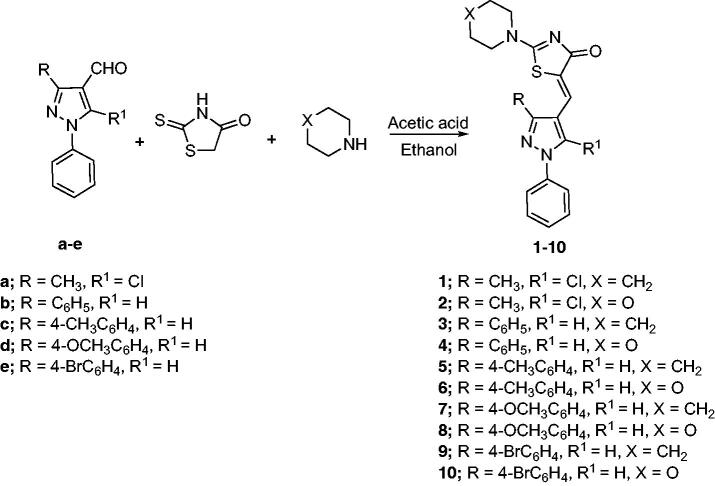
Synthesis of the target compounds **1–10**

The synthetic strategy adopted for the preparation of the target compounds is outlined in Scheme 1. The starting aldehydes were prepared as previously reported^47–51^. The target compounds **1–10** were attained by a one-pot reaction of the aldehydes **a-e** with rhodanine and cyclic secondary amines (namely; piperidine and morpholine) in refluxing ethanol using catalytic amount of acetic acid, similar to previously reported procedure^52^^,^^53^. The reaction proceeded via Knoevenagel condensation of the appropriate aldehyde and rhodanine with subsequent replacement of the sulphur of the thiocarbonyl functionality with secondary amine in the same reaction mixture. The secondary amine has a dual role by acting as the catalyst for the Knoevenagel condensation and behaving as the nucleophile in the next step. It is necessary to point out that compounds **3** and **4** were previously reported by different synthetic route instead of the one-pot reaction^32^.

The ^1^H-NMR spectra of target compounds **1-10** displayed only one type of methine proton indicating Z-configuration in light of the crystal structures of previously reported analogous compounds^54^^,^^55^. For piperidine derivatives (**1,3,5,7** and **9),** the ^1^H-NMR spectra revealed the characteristic multiplets for C_3,4,5_ and C_2,6_ of the piperidine moiety at the range of 1.58–1.70 and 3.51–3.92 ppm, respectively. Whereas, for the morpholine derivatives (**2,4,6,8** and **10),** the ^1^H-NMR revealed the characteristic multiplet peaks for C_2,6_ and C_3,5_ of the morpholine moiety resonating at the range of 3.61–3.75 and 3.74–3.94 ppm, respectively. Moreover, the ^13 ^C-NMR of all target compounds showed the characteristic signal assigned for the C = O carbon of the thiazolone ring around 179.5 ppm. Also, existence of the same carbonyl was confirmed by the characteristic IR band at 1670.35-1685.79 cm^−1^. Furthermore, thiazolone-C_2_ and methine carbons characteristic peaks appeared around 173.5 and 153.5 ppm, respectively, in the ^13 ^C-NMR spectra.

### 3.2. Biological evaluation

#### 3.2.1. In vitro COX-1/2 and 15-LOX inhibition assays

We used ovine COX-1/human recombinant COX-2 assay kit (Catalog no. 560131; Cayman Chemicals Inc. Ann Arbour, MI, USA) to test the *in vitro* COX-1/COX-2 inhibitory activities of the synthesised compounds. IC_50_ values (μM) and selectivity indices (SI) were calculated. Quercetin (selective 12/15-LOX inhibitor), nordihydroguaiaretic acid (NDGA, universal LOX inhibitor) and the LOX inhibitory drug meclofenamate, were used as positive controls for LOX inhibition assay. Celecoxib (selective COX-2 inhibitor), diclofenac and indomethacin (non-selective COX inhibitors) were used as references for COX inhibition assay.

Generally speaking, as depicted in Table 1, all compounds showed submicromolar IC_50_ values for COX-2 inhibition along with being one to two orders of magnitude lower than those for COX-1 inhibition. They were both more active and selective than the reference drugs diclofenac and indomethacin as COX-2 inhibitors. For 15-LOX inhibition, IC_50_ values for all compounds operated in the one-digit micromolar range and were more potent than both meclofenamate and NDGA. Additionally, compounds **1-3,6** and **7** were either more potent or equipotent to quercetin.

**Table 1.** *In vitro* COX-1/2, 15-LOX inhibition IC_50_ values and COX selectivity indices of the newly synthesised compounds

Code	Structure	IC_50_ (μM)^a^			SI^b^ (COX-1/ COX-2)
		15-LOX	COX-1	COX-2	
Celecoxib	—	nd^c^	14.8	0.05	296
Diclofenac Na	—	nd	3.9	0.8	4
Indomethacin	—	nd	0.039	0.49	0.08
Meclofenamate Na	—	5.64	nd	nd	nd
Quercetin	—	3.34	nd	nd	nd
NDGA	—	10.56	nd	nd	nd
1	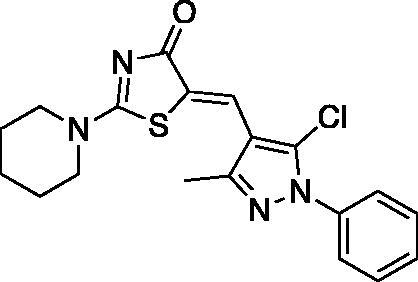	1.96	6.56	0.11	59
2	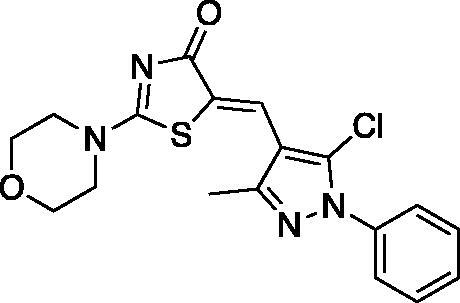	2.42	8.14	0.09	90
3	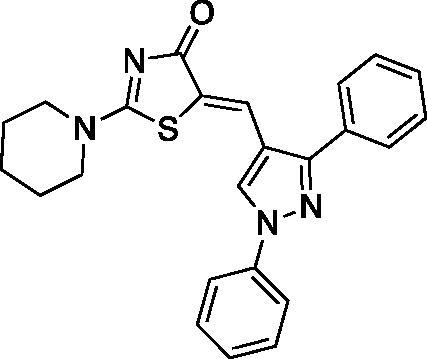	2.54	7.86	0.19	41
4	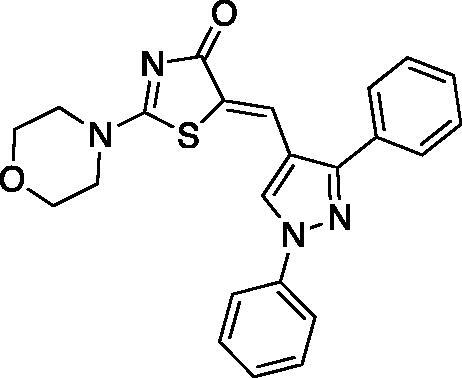	4.63	11.32	0.14	80
5	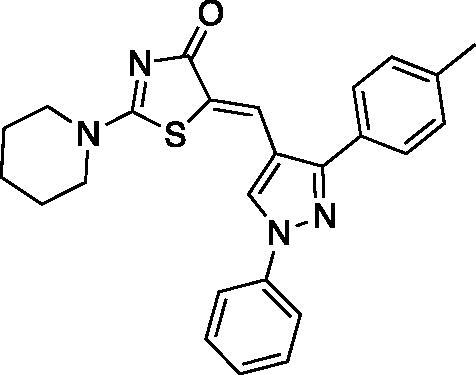	4.23	9.23	0.34	27
6	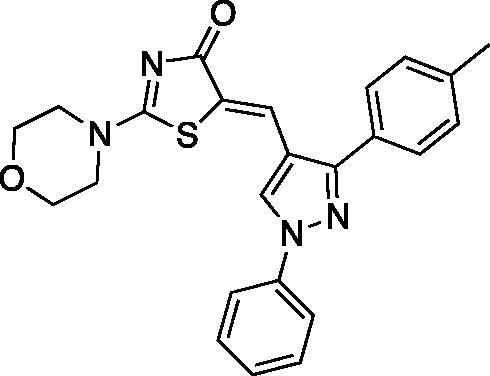	2.85	12.54	0.18	69
7	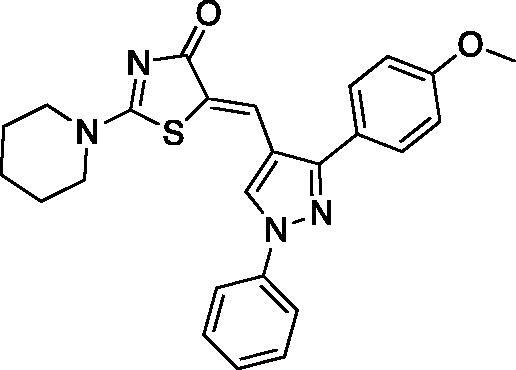	3.52	10.34	0.09	114
8	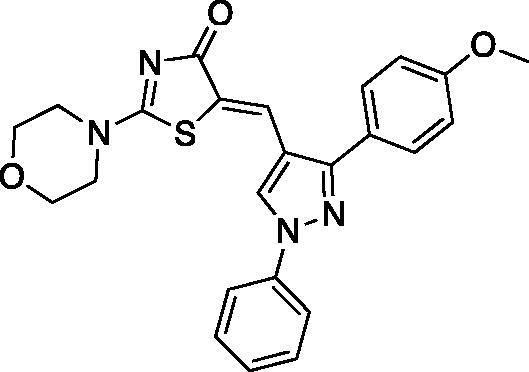	4.64	12.45	0.11	113
9	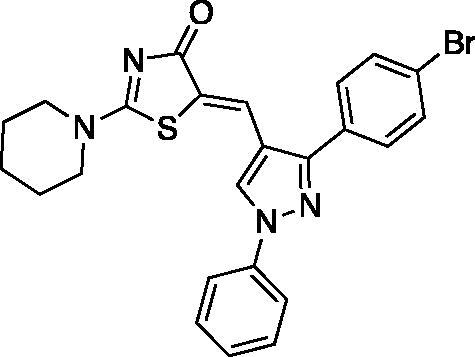	5.64	14.02	0.13	107
10	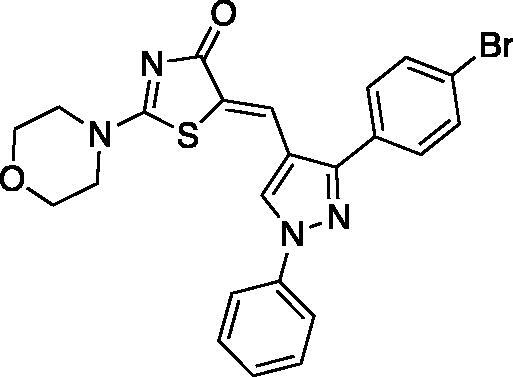	4.52	11.33	0.11	103

^a^IC_50_ = concentration, in micromolar, that causes 50% inhibition of COX-1, COX-2 and 15-LOX enzymatic activity. Values are shown as mean of three determinations with standard deviation of less than 10% of the mean.

^b^Selectivity index (SI) = IC_50_ (COX-1)/IC_50_ (COX-2).

^c^nd = not determined.

Regarding thiazolones **(1** and **2)** originating from 5-chloropyrazole-aldehyde (**a**), the piperidino-derivative **1** showed 1.7 times the activity of quercetin towards 15-LOX inhibition. Switching to the morpholino analogue **2** slightly decreased 15-LOX inhibitory activity but still spanning within the same order of magnitude. Concerning thiazolones **(3** and **4)** derived from 1,3-diphenyl pyrazole-aldehyde (**b**), piperidine derivative **3** demonstrated higher 15-LOX inhibition that somewhat decreased upon replacement with morpholine moiety, yet, retaining the same one-digit micromolar range of inhibition. As for thiazolones **(5** and **6)** obtained from 1-phenyl-3-*p*-tolyl pyrazole-aldehyde (**c**), the lowest activity for 15-LOX inhibition was noticed with the piperidine derivative **5**. Enhancement of activity occurred upon switching to the morpholino-analogue **6**, showing IC_50_ value of 2.85 μM for 15-LOX inhibition. While in case of the thiazolones **(7** and **8)** bearing an EDG methoxy group on the pyrazole-aldehyde moiety, the piperidine analogue **7** was almost equipotent to quercetin for 15-LOX inhibition but the activity decreased upon shifting to the morpholine derivative **8**. Finally, the thiazolones **(9** and **10)** carrying bromo substituent on the pyrazole-aldehyde moiety had 15-LOX inhibitory activities corresponding to 59% and 74% of the activity of quercetin, respectively.

As for COX-2 inhibition, careful inspection of the results revealed no appreciable differences in the IC_50_ values (0.09–0.19 μM) of the synthesised compounds, with the exception of compound 5 (IC_50_ value of 0.34 μM). This is in addition to some unique structural difference between the synthesised compounds, which impeded extracting sharper structure–activity relationships. However, the outcome of this study represents a good starting point to attempt a wider variety of substitution patterns that might aid in establishing decisive structure-activity relationships. However, and with regards to COX selectivity indices, it was clearly evident that thiazolones (**7-10),** bearing methoxy or bromo substitution, showed the highest selectivity (103-114). While for thiazolones (**1-6)**, a decreased selectivity was generally observed with SI values ranging from 27-90. Among the latter, the morpholine derivatives **2**, **4** and **6** demonstrated superior selectivity to the piperidine derivatives **1**, **3** and **5**.

#### 3.2.2. In vivo anti-inflammatory activity

**Figure 2.**
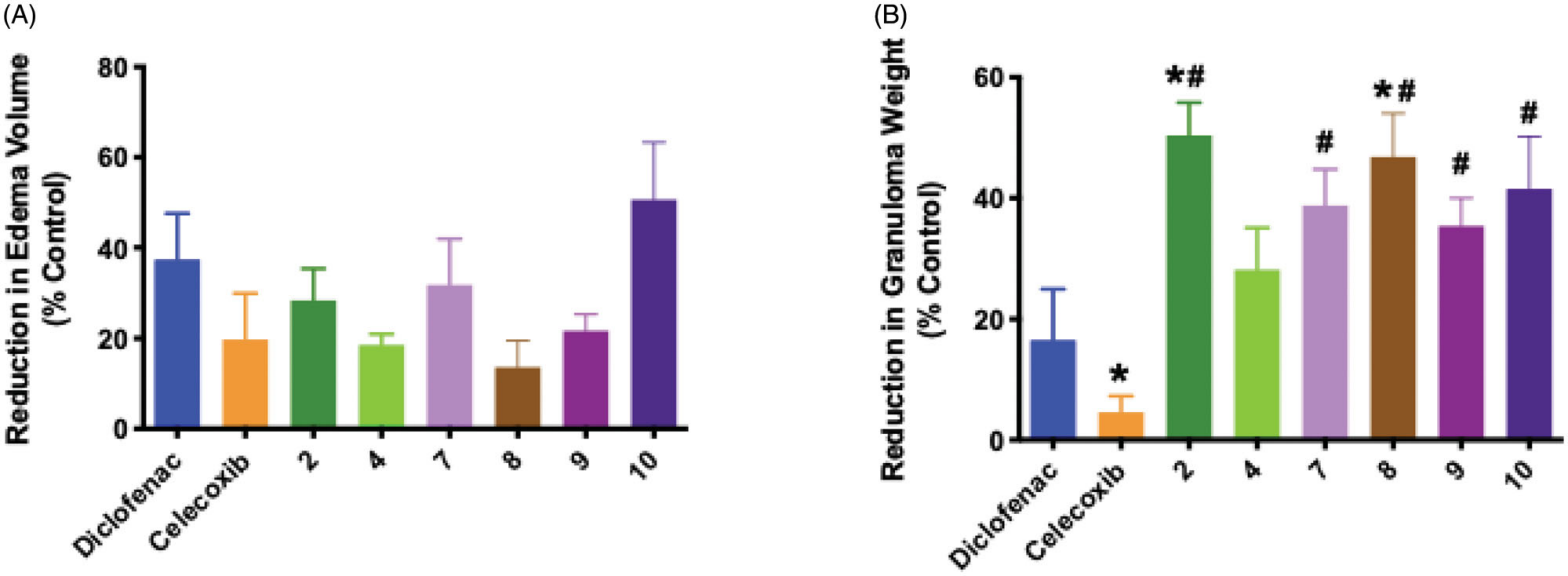
*In vivo* anti-inflammatory activities of compounds **(2,4,7–10)** in formalin-induced rat paw edoema assay (model of acute inflammation) (**A**) and in cotton pellet induced granuloma test (model of chronic inflammation) (**B**). Statistical significance was assessed by one-way ANOVA followed by Tukey multiple comparisons test. * and ^#^ denote *p* values < 0.05 vs. diclofenac and celecoxib, respectively.

The *in vivo* anti-inflammatory activity of the six most active compounds in the *in vitro* COX and LOX inhibition assays **(2,4,7-10)** were challenged using the acute inflammation model formalin-induced rat paw oedema bioassay. Inflammation was stimulated by subcutaneous injection of formalin and the test compounds were administered as an oral dose of 5 mg/kg body weight. The % inhibition of oedema after 4 h was measured to determine potencies of the test compounds in comparison to the control. The positive controls used were celecoxib and diclofenac sodium. Results indicated that compounds **(2,7,9,10)** were as effective as either diclofenac or celecoxib in suppressing acute inflammation measured by inhibition of formalin-induced rat paw oedema (Figure 2(A)).

While, in the cotton pellet induced-granuloma test used as a chronic model of inflammation, all tested compounds demonstrated clear anti-inflammatory activity of a magnitude equal or superior to reference compounds (Figure 2(B)**).** Specifically, compounds **(2 and 8)** showed increased inhibition of granuloma weight in comparison to both celecoxib and diclofenac, whereas the activities of compound **(7,9,10)** were only superior to celecoxib.

#### 3.2.3. Gastric ulcerogenic activity and histopathological examination:

**Figure 3.**
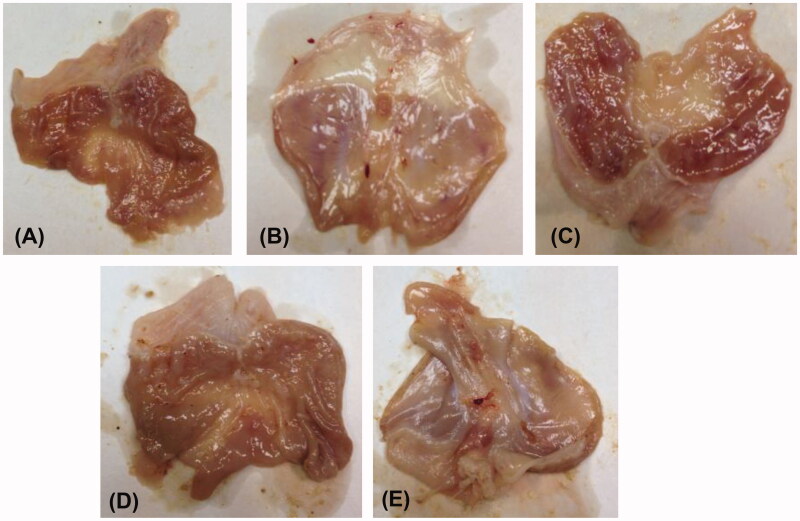
Gross examination of the isolated rat stomachs. Panel **A** shows normal texture demonstrated by **2.** Panels (**B** and **C**) and (**D** and **E**) show variable levels of hyperaemia without gross ulceration by **4** and **10**, respectively.

**Figure 4.**
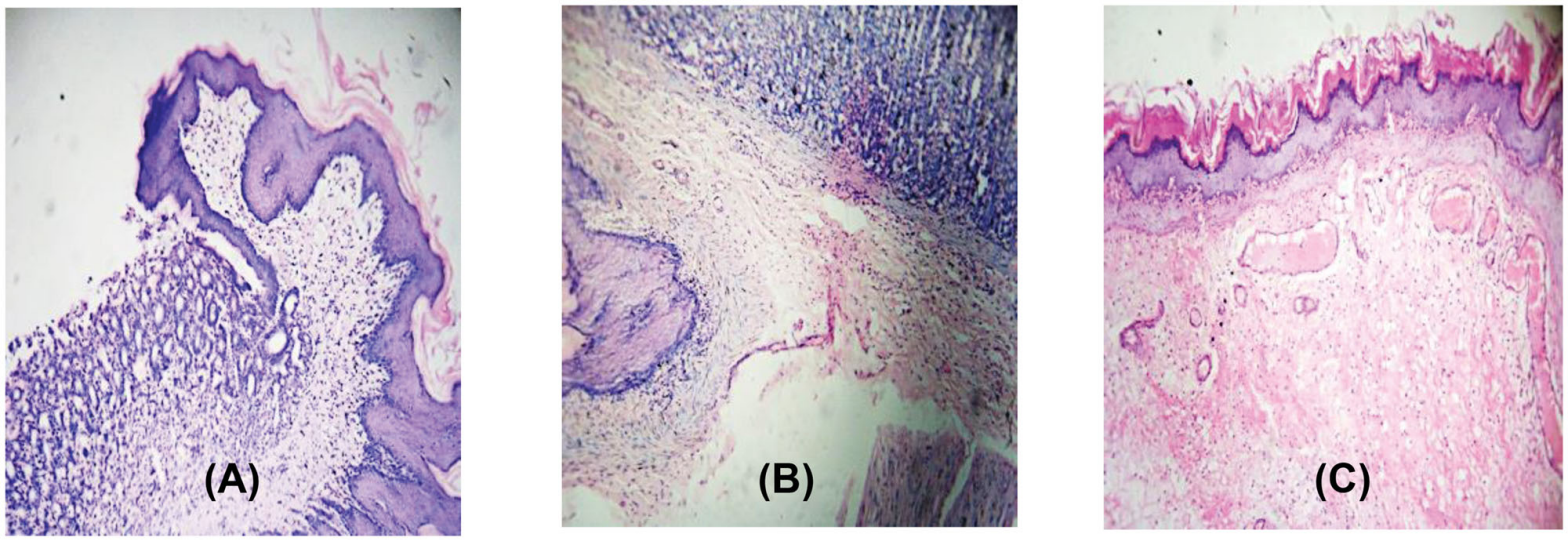
Light microscopic shots of rats’ gastric mucosa treated with compounds **2**, **4** and **10** (H&E 100 x). Panel **A** showed no ulceration with normal gastro-esophageal junction exemplified by **2**. Panel **B** displayed gastro-esophageal inflammatory infiltrates exhibited by **4**. Panel **C** revealed both edoema and inflammatory changes in stomach mucosal surface produced by **10**.

The same six compounds were further examined for their ulcerogenic liability in rats. Gross observation of the isolated rat stomachs demonstrated a normal stomach texture for compounds (**2** and **7-9)** in addition to the reference celecoxib and diclofenac sodium as well as DMSO-treated groups (Figure 3(A)). While for compounds 4 (Figure 3(B,C)) and 10 (Figure 3(D,E)), variable levels of hyperaemia without gross ulceration were detected. Moreover, the histopathological examination of the degree of inflammatory reaction in the stomach gastric layers revealed superior gastrointestinal safety profile for compounds (**2** and **7-9)** (no ulceration with normal gastro-esophageal junction) as well as the references celecoxib and DMSO negative control. Figure 4(A) illustrated the effect of compound **2** as representative example of the safest compounds. On the other hand, as expected from the gross observation, compound **4** exhibited esophago-gastric inflammation (Figure 4(B)) and compound **10** showed both edoema and inflammatory changes (Figure 4(C)).

#### 3.2.4. Inhibition of monocyte-to-macrophage differentiation assay

**Figure 5.**
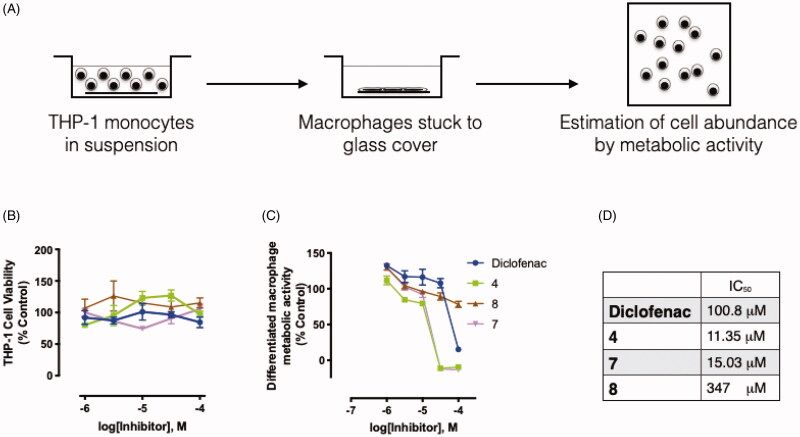
The effect of selected compounds on the differentiation of THP-1 monocytes to the pro-inflammatory M1 macrophage polarisation. Panel **A** shows a schematic for the assay performed, panel **B** depicts the THP-1 cell viability at the different drug concentrations used for the assay, while panel **C** shows the concentration-dependent inhibition of monocyte differentiation into macrophage in response to sequential stimulation with PMA and LPS. The calculated IC_50_ values are listed in **D**.

Following the *in vivo* assays demonstrating equal or superior anti-inflammatory activities of the tested compounds, we assessed their cellular effects on the pro-inflammatory M1 polarisation of macrophages. Three compounds were selected for this assay with a variety of dual COX-2 and LOX inhibitory activities, relatively low gastric ulcerogenic activity, and a range of *in vivo* anti-inflammatory activities. Compounds **4**, **7** and **8** were chosen to represent compounds with *in vivo* activities higher than both diclofenac and celecoxib, higher than celecoxib, or equivalent to both reference compounds, respectively. For the purpose of monitoring the effect on monocyte recruitment and subsequent activation into macrophages, we utilised the PMA-induced THP-1 differentiation assay as a typical *in vitro* model of this process^56^. We tested the inhibitory effects of the compounds on monocyte differentiation into pro-inflammatory M1 macrophages, using PMA and LPS consecutively for THP-1 stimulation^57^ (Figure 5). The effects of these dual inhibitors were compared to diclofenac as a reference compound. The range of concentrations used for the assay was shown not to induce any cytotoxicity on THP-1 cells (Figure 5(B)**).** As shown in Figure 5(C), all three compounds inhibited THP-1 differentiation into M1 macrophages in a concentration-dependent manner. Calculated IC_50_ values are shown in the table (Figure 5(D)**)** with **4** and **7** demonstrating higher potency compared to diclofenac. Moreover, we tested the inhibitory effects of the compounds on monocyte differentiation into resting M0 macrophages, upon stimulation of THP-1 cells with PMA only. Interestingly, while diclofenac showed a comparable IC_50_ to that observed for the M1 macrophage differentiation, the tested compounds showed at least 6-fold less potency towards the resting polarisation (data not shown). Not only do these results support the anti-inflammatory potential of these inhibitors, they provide evidence that our tested compounds may exhibit a more biased effect towards inhibition of differentiation into pro-inflammatory macrophages.

#### 3.2.5. IL-1β expression

Previous studies have demonstrated an association between 15-LOX activation and cytokine production in multiple cell lines^58–60^. In particular, 15-LOX and its metabolites were shown to induce the production of a number of proinflammatory cytokines in macrophages, including tumour necrosis factor-α (TNF-α), IL-1β, IL-6, IL-12, and monocyte chemo-attractant protein-1^61^^,^^62^. The produced IL-1β causes further activation of a signalling pathway leading to the enhancement of phospholipase A_2_-dependent arachidonate release and metabolism in a positive feedback loop^63^. Interestingly, 15-LOX expression in macrophages also contributes to atherosclerosis progression, via enhancing lipid accumulation and cytokine production^64^. Furthermore, a body of evidence has proposed a role for COX-2 in increasing the production of the pro-inflammatory cytokine IL-1β. PGE_2_, a major COX-2 metabolite, enhanced NLRP3 inflammasome-dependent IL-1β production in macrophages through enhancing cAMP levels^65^^,^^66^. In this context, various studies demonstrated that COX-2 inhibition abolished the expression of IL-1β^67^^,^^68^.

**Figure 6.**
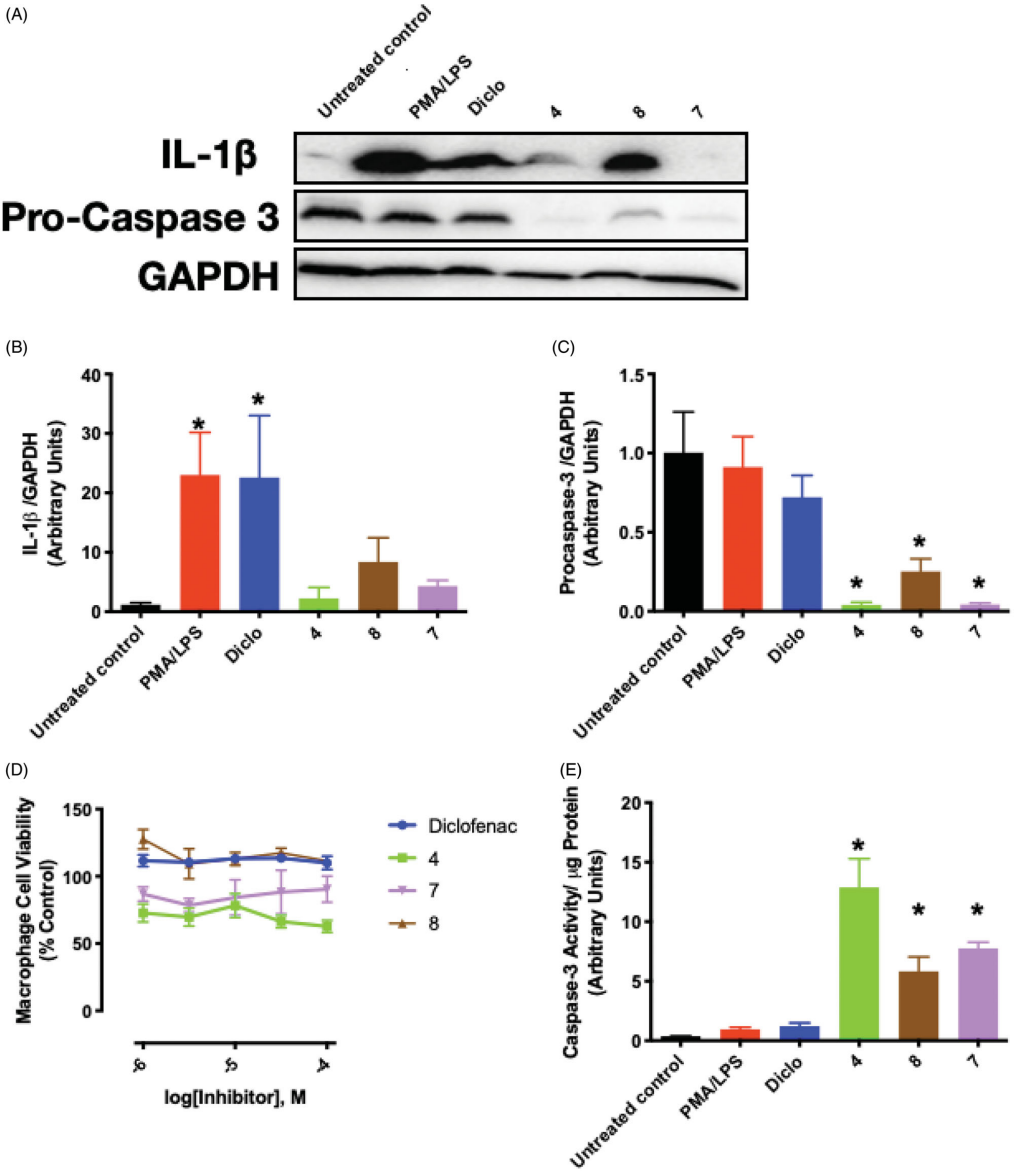
The effect of selected compounds on IL-1β expression and apoptotic changes in M1 macrophages. Panel **A** shows representative blots for IL-1β and procaspase-3, panels **B & C** depict the quantified protein expression levels for IL-1β and procaspase-3, respectively, panel **D** demonstrates M1 macrophage viability at the different drug concentrations used, and panel **E** represents the increased caspase-3 activity in M1 macrophages treated with different compounds. Statistical significance was assessed by one-way ANOVA followed by Tukey multiple comparisons test. * and ^#^ denote *p* values < 0.05 vs. untreated control and PMA/LPS differentiated macrophages, respectively.

In an attempt to provide a pathophysiological context for the dual COX-2/15-LOX inhibition, we tested the effect of our hybrid compounds on IL-1β expression in THP-1 monocytes challenged with PMA and LPS (Figure 6). PMA/LPS treatment exhibited a marked increase in IL-1β production when compared to untreated control. Such an increase in IL-1β expression was significantly attenuated in cells treated with our compounds (Figure 6(A,B)).

#### 3.2.6. Induction of macrophage apoptosis

Although our dual COX-2/15-LOX inhibitors did not demonstrate cytotoxic effects on THP-1 monocytes up to the highest concentration used (100 μM), **4** and **7** tended to slightly decrease PMA/LPS-stimulated THP-1 (M1 macrophages) cell viability (Figure 6(D)**)**. In view of the enhanced inhibitory effects of the compounds on M1 macrophages, such an observation proposed a possible cell death mechanism induced by our dual inhibitors. Along these lines, non-steroidal anti-inflammatory drugs were previously reported to manifest apoptotic effects in peritoneal macrophages via induction of CHOP, an ER stress response-related protein^69^. Specifically, EP4-independent PGE_2_ signalling was shown to promote macrophage apoptosis and attenuate early inflammatory changes^70^. Therefore, we examined the protein expression and activity of caspase-3, a key regulator of apoptosis. Our hybrid compounds attenuated the protein expression of non-cleaved procaspase-3, hence suggesting an enhanced cleavage of procaspase-3 into its active form caspase-3 (Figure 6(A,C)). Consistently, these dual inhibitors also produced a significant enhancement of caspase-3 activity in PMA/LPS-induced THP-1 monocytes (Figure 6(E)).

### 3.3. Molecular modelling and in silico studies

#### 3.3.1. Molecular docking study on COX-2 enzyme

For better understanding of the interaction of our compounds with their biological target at the molecular level, we docked 2 representative examples which are **4** and **7** into COX-2 active site and subsequently examined their binding modes. Docking experiments were performed using Molecular Operating Environment (MOE) version 2016.0802 (Chemical Computing Group, Montreal, CA) and the protein data bank file 1CX2. Validation via redocking of the co-crystallised ligand yielded a pose retrieval with RMSD less than 1 Å (Figure SM1, Supplementary Material) which confirms the suitability of the docking protocol. In order to gauge the binding affinities to the COX-2 active site, we determined the docking scores, hydrogen bonds established with the neighbouring amino acids, and spatial orientation of the docked compounds in comparison to the native ligand SC-558. Generally speaking, both compounds fitted perfectly into the active site in a similar pattern to that of the co-crystallised ligand SC-558.

**Figure 7.**
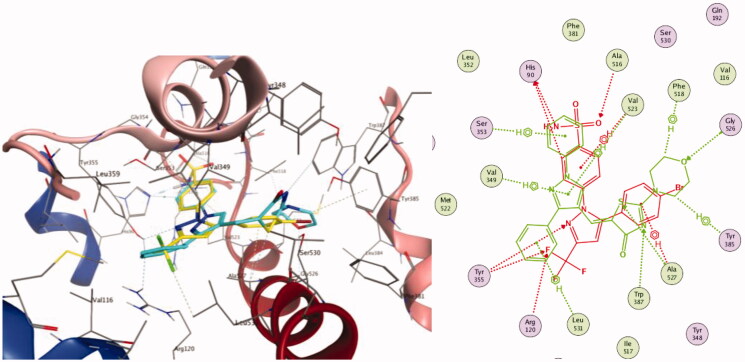
A comparison between the docked pose of compound **4** (in green for 2 D and cyan for 3 D) with the co-crystallised ligand SC558 (in red for 2 D and yellow for 3 D). The right and left panels are the overlay of both poses in 2 D and 3 D views, respectively.

Inspection of the most favourable pose of compound **4** in complex with COX-2 enzyme highlighted its similar orientation to that of the co-crystallised ligand (Figure 7). It was lodged in the active site through three hydrogen bonds. Of particular interest, the morpholine oxygen was hydrogen bonded to Gly526 (distance of 3.32 Å). The thiazolone ring participated in 2 hydrogen bonds through its Nitrogen and Sulphur atoms with Trp387 (4 Å) and Ala527 (3.33 Å) residues, respectively. The complex was further stabilised via six arene-hydrogen interactions; two of them were between the morpholine ring and Tyr385 and Phe518, another two were between the pyrazole ring and Val349 and Val523, along with two more hydrophobic contact between the 2 phenyl rings and Ser353 and Leu531.

**Figure 8.**
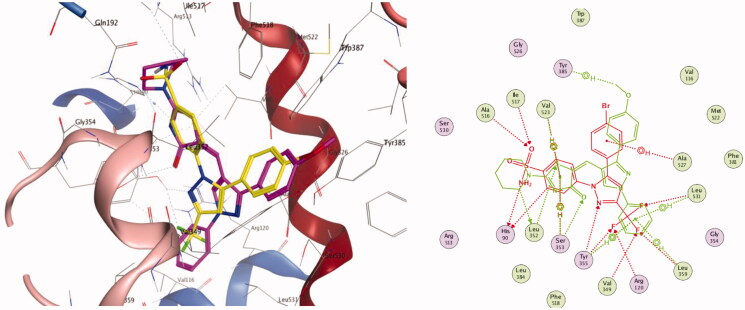
A comparison between the docked pose of compound **7** (in pink for 3 D and green for 2 D) with the co-crystallised ligand SC558 (in yellow for 3 D and red for 2 D). The right and left panels are the overlay of both poses in 2 D and 3 D views, respectively.

For the piperidine derivative **7,** it also adopted a binding mode similar to that of SC-558 (Figure 8). It was perfectly anchored in the active site cavity through three hydrogen bonds; two of them existed between the thiazolone ring and Leu352 and Ser353 (distance of 4.24 and 3.02 Å, respectively). This is in addition to two arene-hydrogen contacts between the thiazolone ring and Ser353 and Val523. The interaction pattern of the thiazolone ring was also spotted with compound **7**, which emphasises its part in biomolecular target recognition. Moreover, the phenyl ring contributed three arene-hydrogen contacts with Tyr355, Leu359 and Leu531.

#### 3.3.2. Molecular docking study on 15-LOX enzyme

In order to gain insight into the possible binding interactions of the representative compounds **4** and **7** with 15-LOX enzyme, docking experiments of the mentioned compounds were performed using Molecular Operating Environment (MOE) version 2016.0802 (Chemical Computing Group, Montreal, CA) and the protein data bank file 1LOX. Pose retrieval of the co-crystallised ligand RS7 produced an RMSD of 0.44 Å, which validated the adopted docking protocol (Figure SM2, supplementary material).

Again, docking scores, hydrogen bonds established with the surrounding amino acids, and spatial orientation of the docked compounds in comparison to the native ligand RS7 were used to evaluate the binding affinities of our target two compounds.

**Figure 9.**
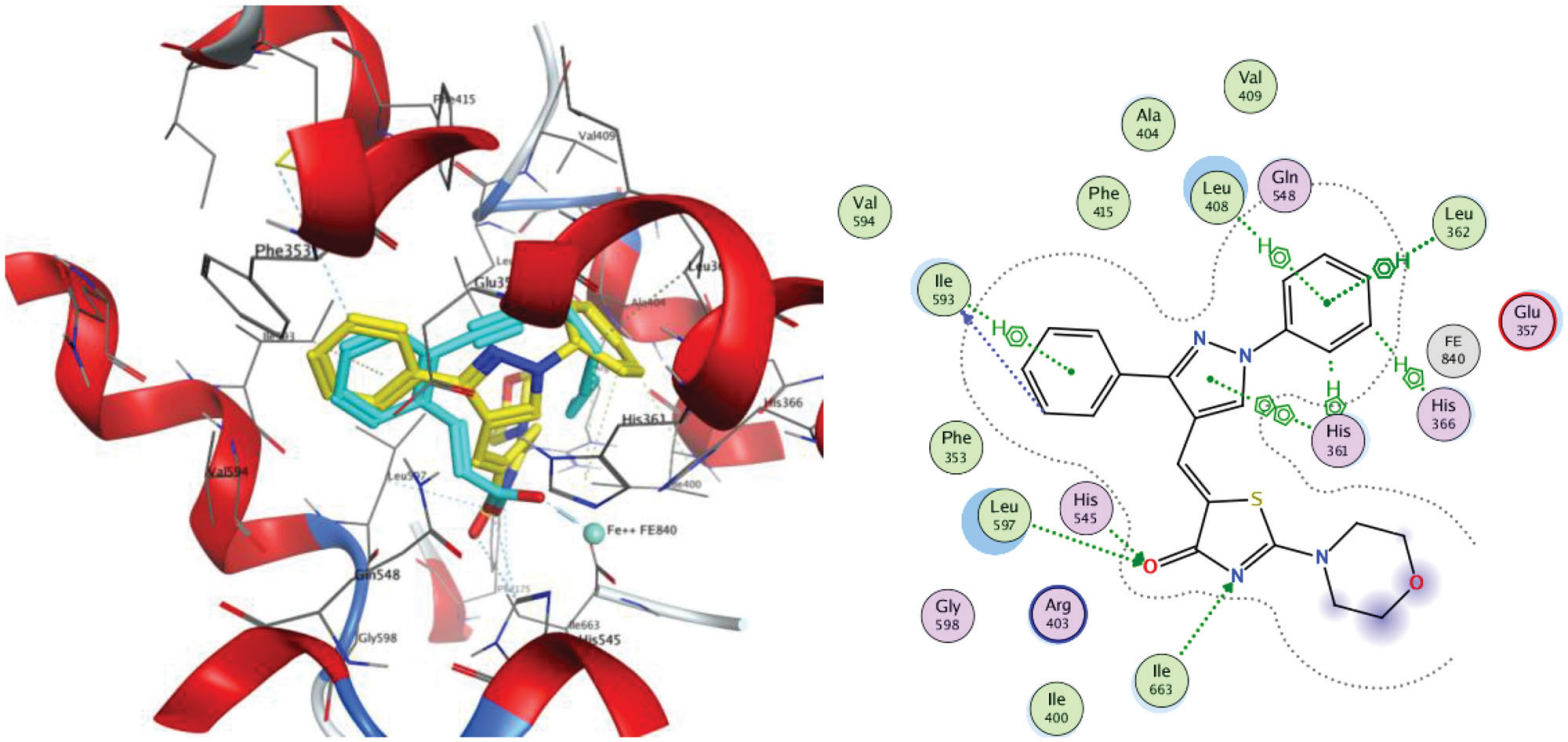
Docking and binding pattern of compound **4** into 15-LOX active site (PDB 1LOX) in 3 D (left panel) and 2 D (right panel). The 3 D pose contains an overlay of **4** (yellow) over the co-crystallised ligand RS7 (cyan).

Regarding compound **4**, three evident H-bonding interactions were noticed with both His 545 and Leu597 (**4** as an acceptor via thiazolone carbonyl, distance of 3.2 Å) and Ile663 (**4** as an acceptor via thiazolone nitrogen, distance of 3.69 Å). Besides, nine hydrophobic interactions were observed with His361, Leu362, His366, Leu408, Ile593, along with pyrazolyl and both phenyl rings (Figure 9).

**Figure 10.**
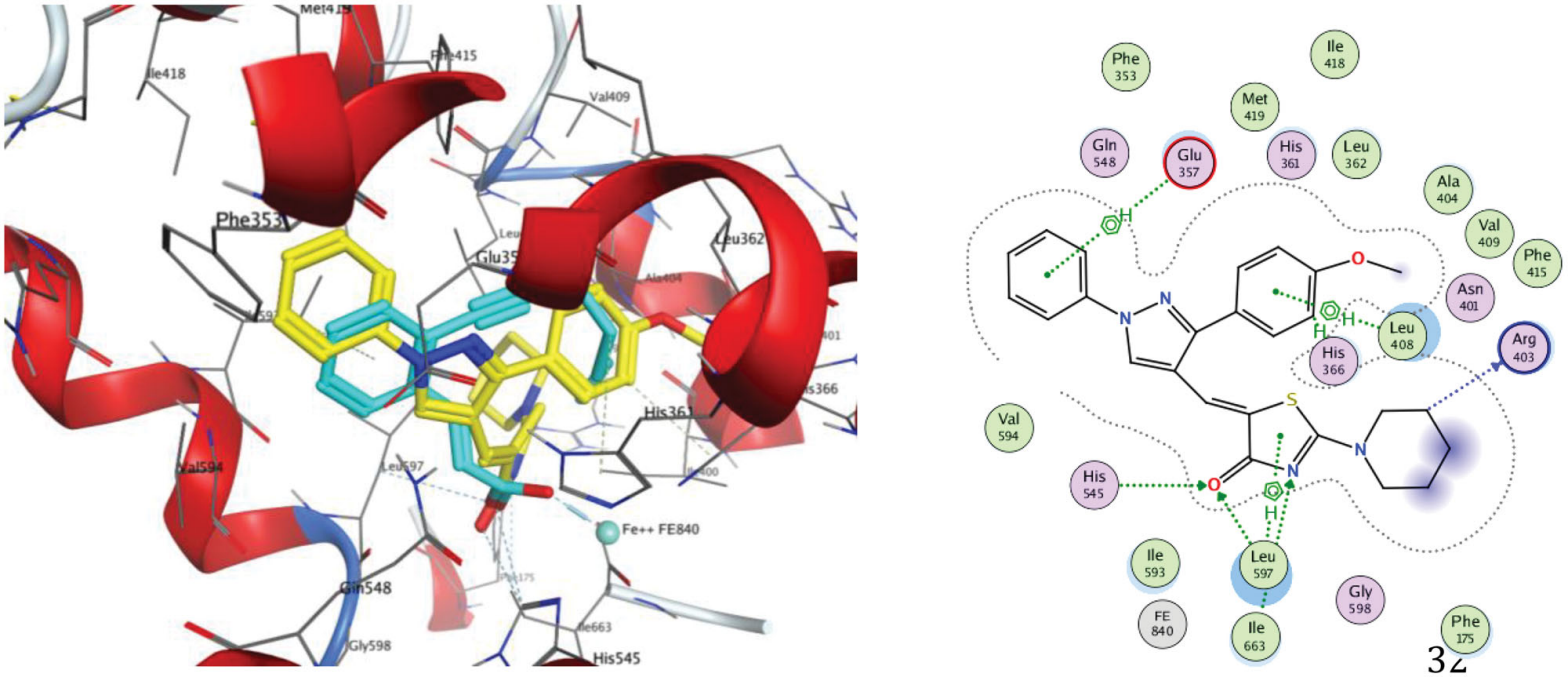
Docking and binding pattern of compound **7** into 15-LOX active site (PDB 1LOX) in 3 D (left panel) and 2 D (right panel). The 3 D pose contains an overlay of **7** (yellow) over the co-crystallised ligand RS7 (cyan).

Concerning Compound **7** (Figure 10), it was perfectly positioned in the active site cavity through three hydrogen bonds, with the following residues: His 545 and Leu597 (**7** as an acceptor via thiazolone carbonyl, distance of 3.3 Å) and Ile663 (**7** as an acceptor via thiazolone nitrogen, distance of 3.77 Å). Moreover, nine hydrophobic interactions were observed with Glu357, Leu408, His366, Leu597, along with thiazolone and both phenyl rings.

As such, the results of the docking studies performed on compounds **4** and **7** could shed light on the molecular basis for their *in vitro* and *in vivo* activity.

#### 3.3.3. In silico estimation of physicochemical properties, drug-likeness and pharmacokinetics

In the present work, we estimated the pharmacokinetic profile, drug-likeness and conformity to Lipinski’s rule and physicochemical properties for the most active six compounds by Molinspiration^43^, Pre-ADMET^44^, ProTox-II^45^ (http://tox.charite.de/protox_II/) and Data warrior^46^ software. Results are summarised in Tables 2 and 3. The compounds obeyed Lipinski's rule of five, and thus should theoretically exhibit satisfactory passive oral absorption. They demonstrated molecular polar surface area (TPSA) values less than 140 Å^2^ thus designating good intestinal absorption and transport. Moreover, they possessed 3 to 5 rotatable bonds and therefore exhibiting moderate to high conformational flexibility. With positive drug-likeness scores, these compounds can be considered as drug-like. They showed extremely high human intestinal absorption values. Besides, they have predicted LD_50_ values of 250–700 mg/kg. They showed medium cell permeability in the human colon adenocarcinoma cell model, displayed low to medium BBB penetration capability and all except **2c** were strongly bound to plasma proteins.

**Table 2.** *In silico* physicochemical parameters and drug-likeness data of compounds (**2,4,7–10**)

Comp. ID	LogPa	MW^b^	HAc	HD ^d^	Lipinski's violation	TPSA^e^ (A^2^)	Volume (A^3^)	NROTB^f^	Drug likeness
**2**	2.78	388.88	6	0	--	60.26	321.84	3	5.44
**4**	3.43	416.51	6	0	--	60.26	363.15	4	5.14
**7**	4.55	444.56	6	0	--	60.26	396.52	5	4.26
**8**	3.49	446.53	7	0	--	69.5	388.70	5	5.17
**9**	5.3	493.43	5	0	1	51.03	388.86	4	2.49
**10**	4.24	495.40	6	0	--	60.26	381.04	4	3.35

^a^LogP: logarithm of partition coefficient between n-octanol and water. ^b^MW: molecular weight. ^c^HA: number of hydrogen bond acceptors. ^d^HD: number of hydrogen bond donors. ^e^TPSA: topological polar surface area. ^f^NROTB: number of rotatable bonds.

**Table 3.** ADME and toxicity data of the most active compounds.

Comp. ID	Caco2^a^	HIA^b^	BBB^c^	PPB^d^	LD_50_^e^ (mg/kg)
**2**	50.91	98.74	0.25	84.41	700
**4**	32.85	98.08	0.27	93.30	670
**7**	33.45	97.84	0.25	92.66	670
**8**	32.53	98.66	0.31	92.70	250
**9**	49.37	97.78	0.082	99.78	670
**10**	47.01	97.66	0.23	93.69	500

^a^Caco2: permeability through human colon adenocarcinoma cells; Caco2 values < 4 nm/s (low permeability), values ranging from 4 to 70 nm/s (medium permeability) and values > 70 nm/s (high permeability).

^b^HIA: % human intestinal absorption; HIA values ranging from 0 to 20% (low absorption), values from 20 to 70% (moderate absorption) and from 70 to 100% (high absorption). ^c^BBB: blood-brain barrier penetration; BBB values < 0.1 (low CNS absorption), values from 0.1 to 2 (medium CNS absorption) and values > 2 (high CNS absorption). ^d^PPB: plasma protein binding; PPB values < 90% (poorly bound) and values > 90% (strongly bound). ^e^LD_50_: Median lethal dose, Class III: toxic if swallowed (50 < LD_50_ ≤ 300) and Class IV: harmful if swallowed (300 < LD_50_ ≤ 2000).

## 4. Conclusions

As a continuity of our endeavours towards the development of anti-inflammatory agents with minimal ulcerogenic propensities, we are presenting the design and synthesis of a new series of pyrazolyl thiazolones (**1–10**) as dual COX-2/15-LOX inhibitors with potential anti-inflammatory activity. Synthesis proceeded via one pot reaction of substituted pyrazolaldehydes (**a-e**), rhodanine and the appropriate secondary amine. *In vitro* COX inhibition assay data identified seven out of the ten compounds as submicromolar COX-2 inhibitors with IC_50_ values ranging from 0.09 to 0.14 µM, compared to 0.05, 0.8 and 0.49 µM for the reference drugs celecoxib, diclofenac sodium and indomethacin, respectively. In addition, these compounds displayed relatively weak COX-1 inhibitory activities (6.45–14.02 µM). Moreover, six compounds showed reasonable selectivity indices (COX-1/2) of 80-114. 15-LOX inhibitory activities of the test compounds were also assessed. Interestingly, five compounds exerted substantial activities with IC_50_ values spanning from 1.96 to 3.52 µM, compared to 3.34 µM for the reference quercetin. Biological screening results indicated that all compounds showed significant *in vivo* anti-inflammatory activity, equivalent to that of celecoxib and diclofenac in the acute inflammatory model. While compounds **2** and **8** displayed superior anti-inflammatory activity, when compared to diclofenac and celecoxib in the chronic model. As expected, two of these compounds (**4, 7**) demonstrated potent inhibitory effects on monocyte-to-macrophage differentiation, an important and early step in the inflammatory process, with more selective ability to inhibit differentiation into the pro-inflammatory M1 polarisation. Moreover, COX-2/15-LOX inhibitory effects of three compounds (**4, 7, 8)** were manifested by attenuating IL-1β production in M1 macrophages. Interestingly, these compounds also showed apoptotic effects on M1 macrophages, a mechanism that further augments their anti-inflammatory activity. These findings will provide guidance to further chemical modifications for the development of new drug-like clinically useful anti-inflammatory agents.

## Supplementary Material

Supplemental Material
