# Supplementary material for: Challenging inflammatory process at molecular, cellular and in vivo levels via some new pyrazolyl thiazolones
Source: J Enzyme Inhib Med Chem. 2021 Feb 22;36(1):669–84. doi: 10.1080/14756366.2021.1887169 (PMC7901699; doi:10.1080/14756366.2021.1887169)
Supplement: Supplemental Material [file IENZ_A_1887169_SM3663.pdf]

Supplementary Material

Challenging Inflammatory Process at Molecular, Cellular and *In Vivo* Levels Via Some New Pyrazolyl Thiazolones

Perihan A. Elzahhar[1#]*, Rana A. Alaaeddine[2#], Rasha Nassra[3], Azza Ismail[1], Hala F. Labib[4], Mohamed G. Temraz[5], Ahmed S. F. Belal[1]*, Ahmed F. El-Yazbi[2,6]*

[1]*Department of Pharmaceutical Chemistry, Faculty of Pharmacy, Alexandria University, Alexandria 21521, Egypt*
[2]*Department of Pharmacology and Toxicology, Faculty of Medicine and Medical Centre, American University of Beirut, Beirut, Lebanon*
[3]*Department of Medical Biochemistry, Faculty of Medicine, Alexandria University, Alexandria, Egypt*
[4]*Department of Pharmaceutical Chemistry, College of Pharmacy, Arab Academy of Science Technology and Maritime Transport, Alexandria, Egypt.*
[5]*Faculty of Pharmacy, Alexandria University, Alexandria, 21521, Egypt.*
[6]*Department of Pharmacology and Toxicology, Faculty of Pharmacy, Alexandria University, Alexandria 21521, Egypt.*

Contents:

	**Content**	**Page No.**
Figure **SM1**	A comparison between the docked pose of the co-crystallized ligand SC558 that is generated by MOE 2016.0802 with the original one that is deposited in PDB in the active site of COX-2 enzyme (PDB ID 1CX2).	**S2**
Figure **SM2**	A comparison between the docked pose of the co-crystallized ligand RS7 that is generated by MOE 2016.0802 with the original one that is deposited in PDB in the active site of 15-LOX enzyme (PDB ID 1LOX).	**S3**
	^{1}H-NMR and ^{13}C-NMR spectra	**S4-S13**
	Ascertainment of purity by HPLC	**S14-S23**

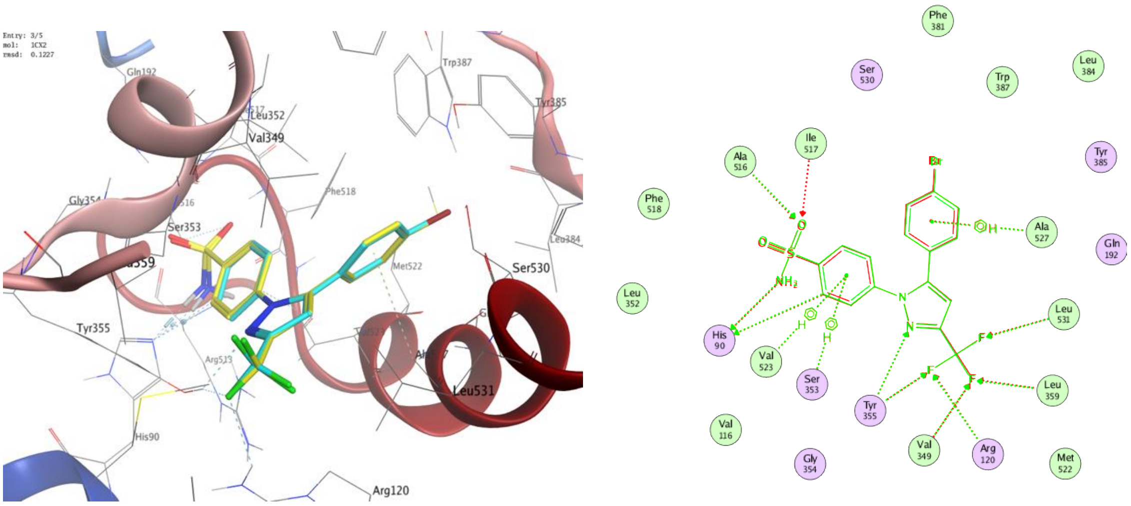

Figure SM1. A comparison between the docked pose of the co-crystallized ligand SC558 (in cyan for 3D and green for 2D) that is generated by MOE 2016.0802 with the original one that is deposited in PDB (in yellow for 3D and red for 2D) in the active site of COX-2 enzyme (PDB ID 1CX2, https://www.rcsb.org/structure/1CX2), with RMSD value of 0.12 Å. The right and left panels are the overlay of both poses in 2D and 3D views, respectively.

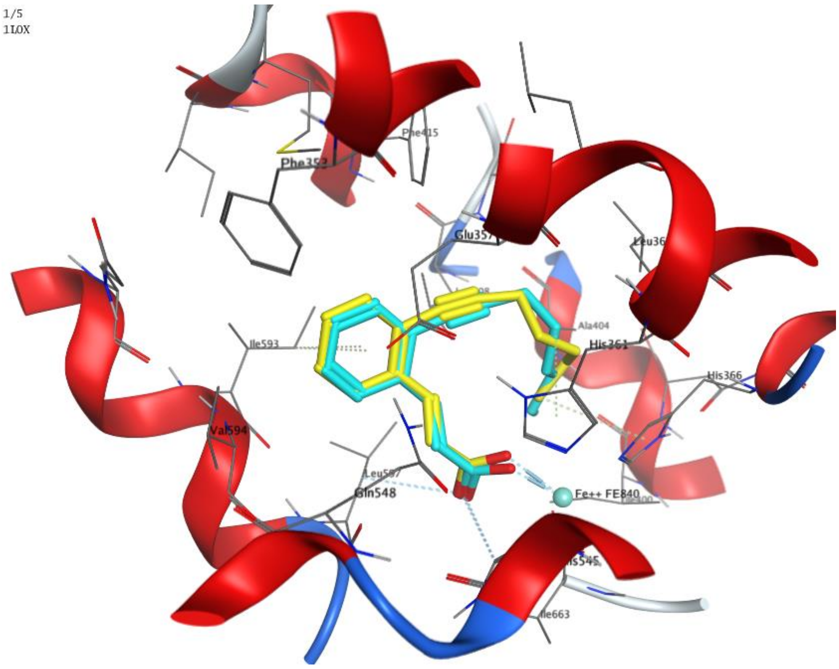

Figure SM2. A comparison between the docked pose of the co-crystallized ligand RS7 (in yellow) that is generated by MOE 2016.0802 with the original one that is deposited in PDB 1LOX (in cyan) in the active site of 15-LOX enzyme (PDB ID 1LOX, https://www.rcsb.org/structure/1LOX), with RMSD value of 0.44 Å.

^{1}H-NMR and ^{13}C-NMR spectra:

(*Z*)-5-((5-chloro-3-methyl-1-phenyl-1*H*-pyrazol-4-yl)methylene)-2-(piperidin-1-yl)thiazol-4(5*H*)-one **(1)**

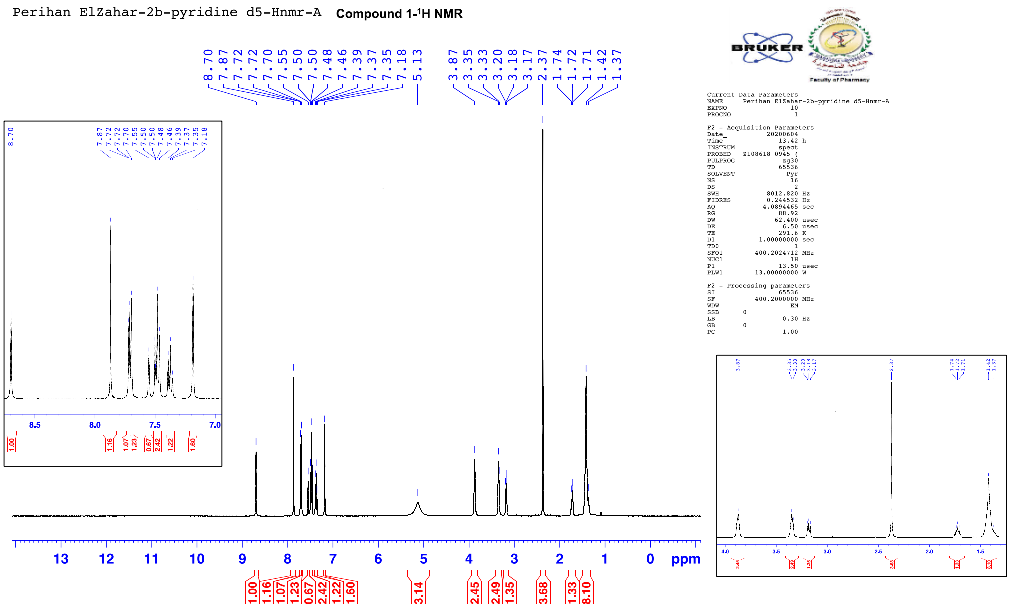

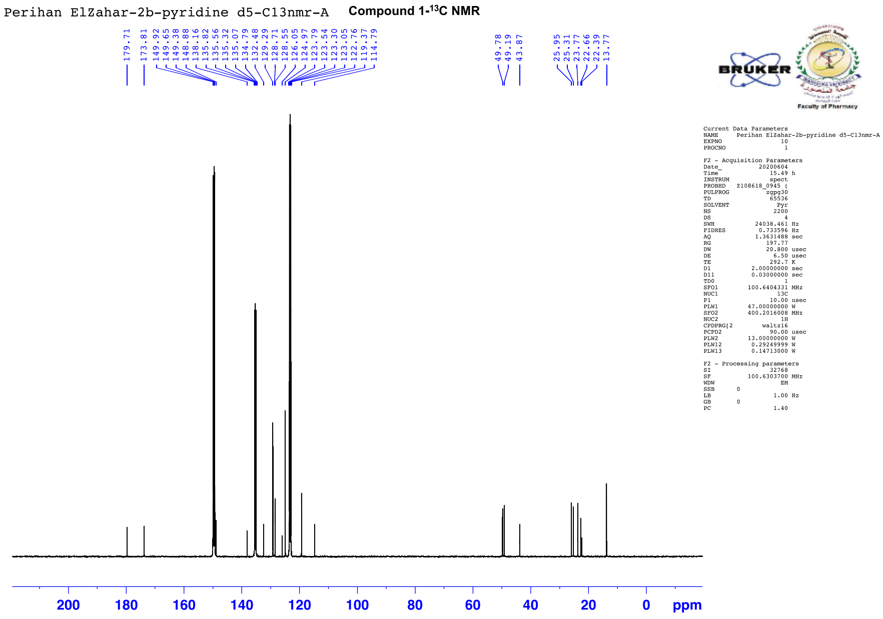

(*Z*)-5-((5-chloro-3-methyl-1-phenyl-1*H*-pyrazol-4-yl)methylene)-2-morpholinothiazol-4(5*H*)-one **(2)**

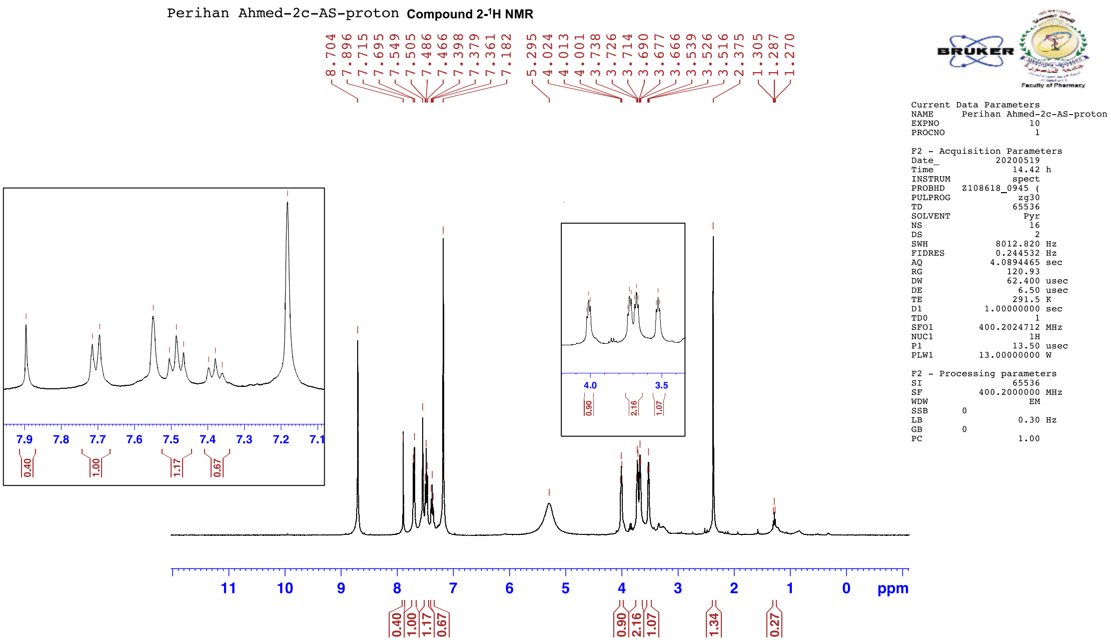

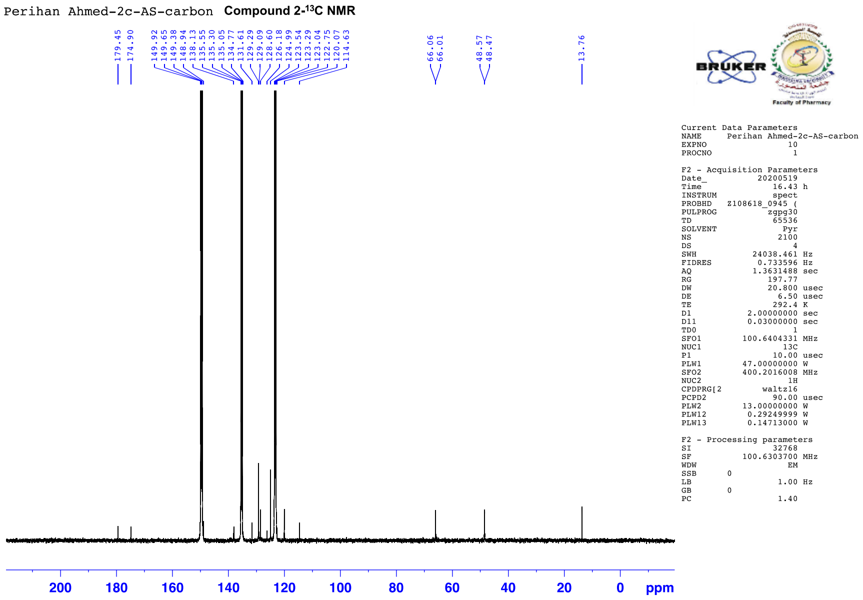

(*Z*)-5-((1,3-diphenyl-1*H*-pyrazol-4-yl)methylene)-2-(piperidin-1-yl)thiazol-4(5*H*)-one **(3)**

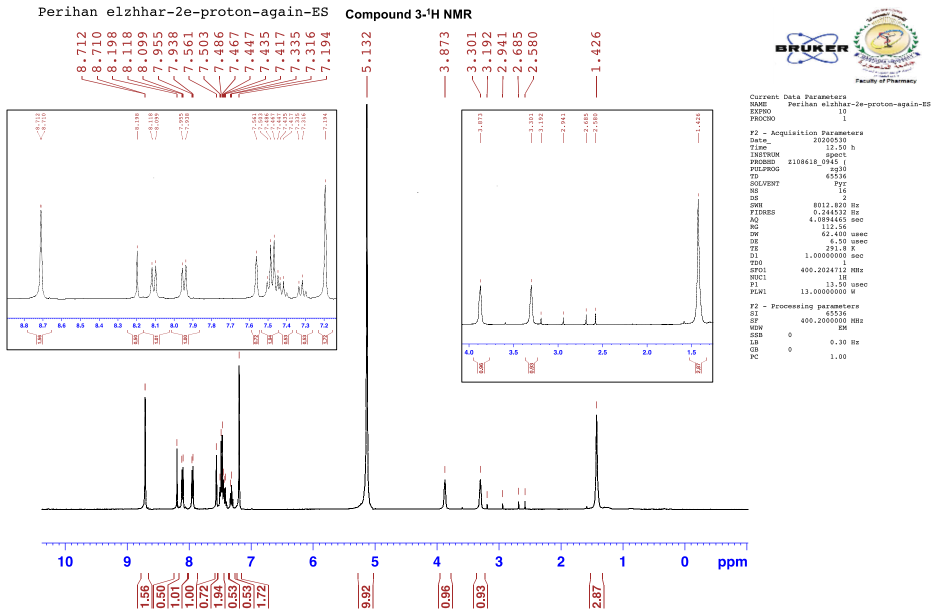

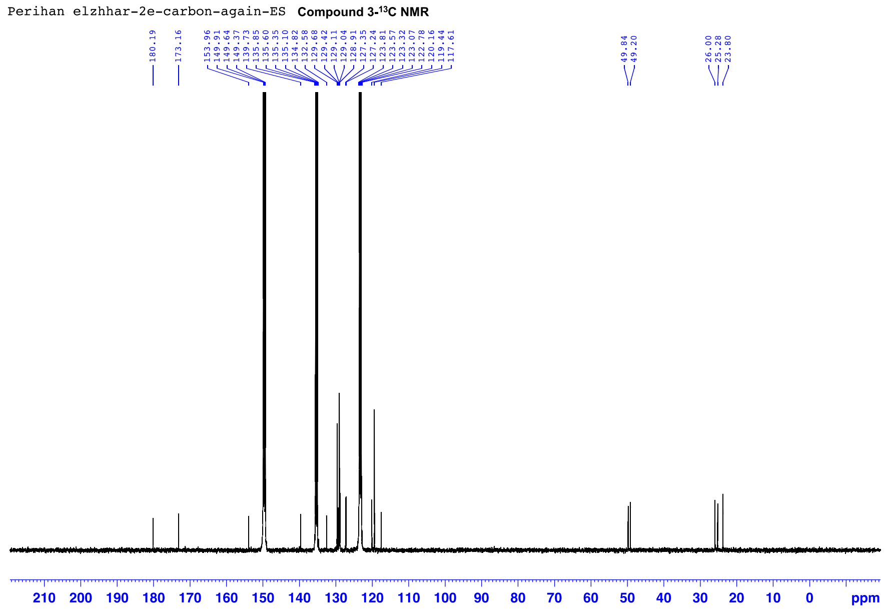

(*Z*)-5-((1,3-diphenyl-1*H*-pyrazol-4-yl)methylene)-2-morpholinothiazol-4(5*H*)-one **(4)**

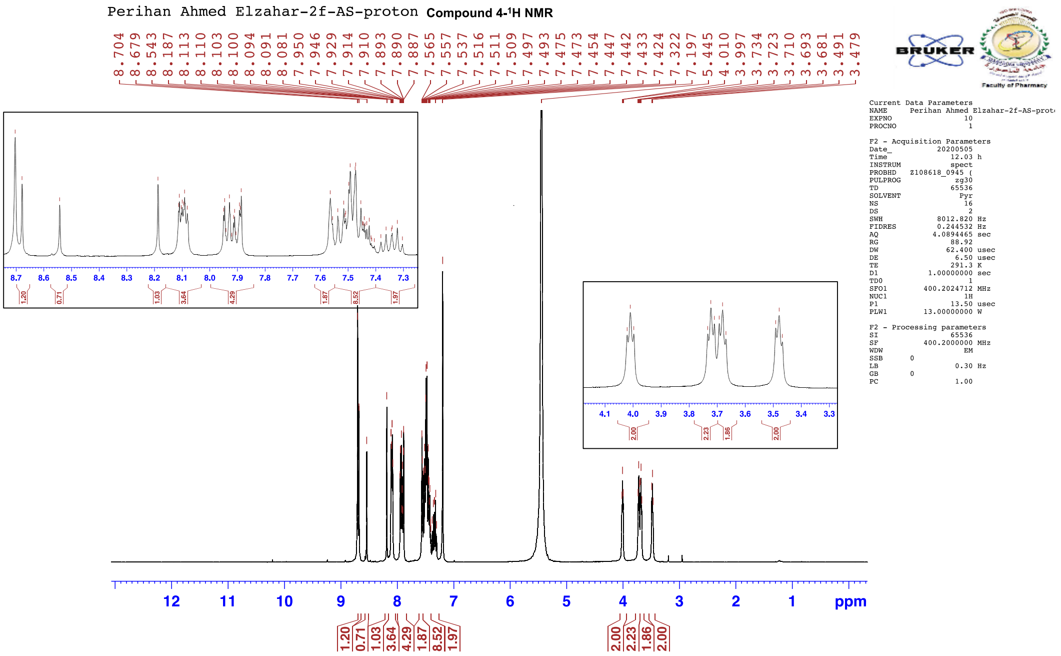

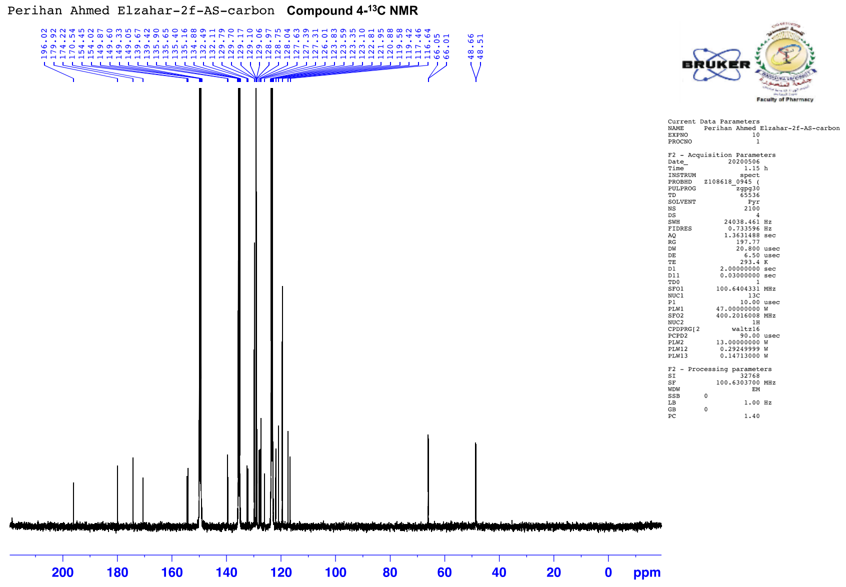

(*Z*)-5-((1-phenyl-3-(*p*-tolyl)-1*H*-pyrazol-4-yl)methylene)-2-(piperidin-1-yl)thiazol-4(5*H*)-one **(5)**

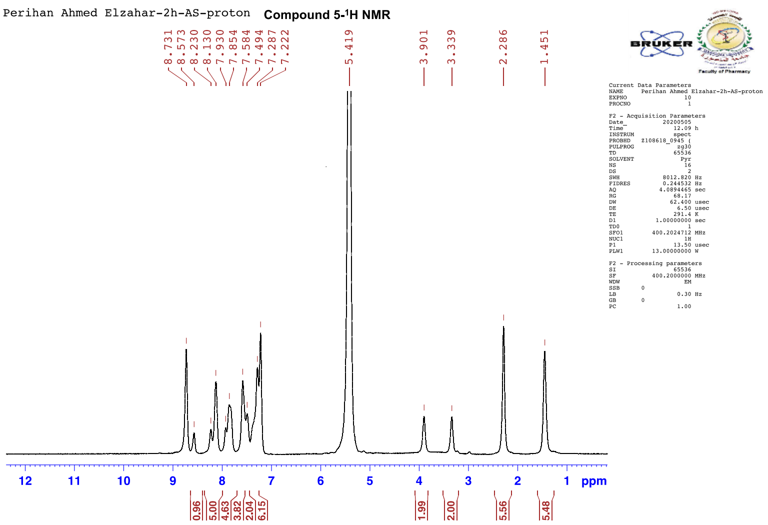

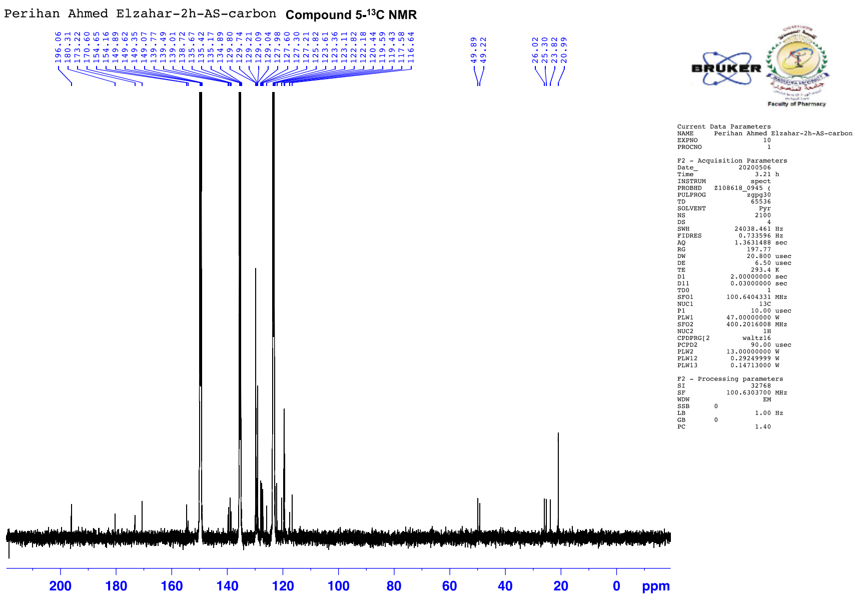

(*Z*)-2-morpholino-5-((1-phenyl-3-(*p*-tolyl)-1*H*-pyrazol-4-yl)methylene)thiazol-4(5*H*)-one **(6)**

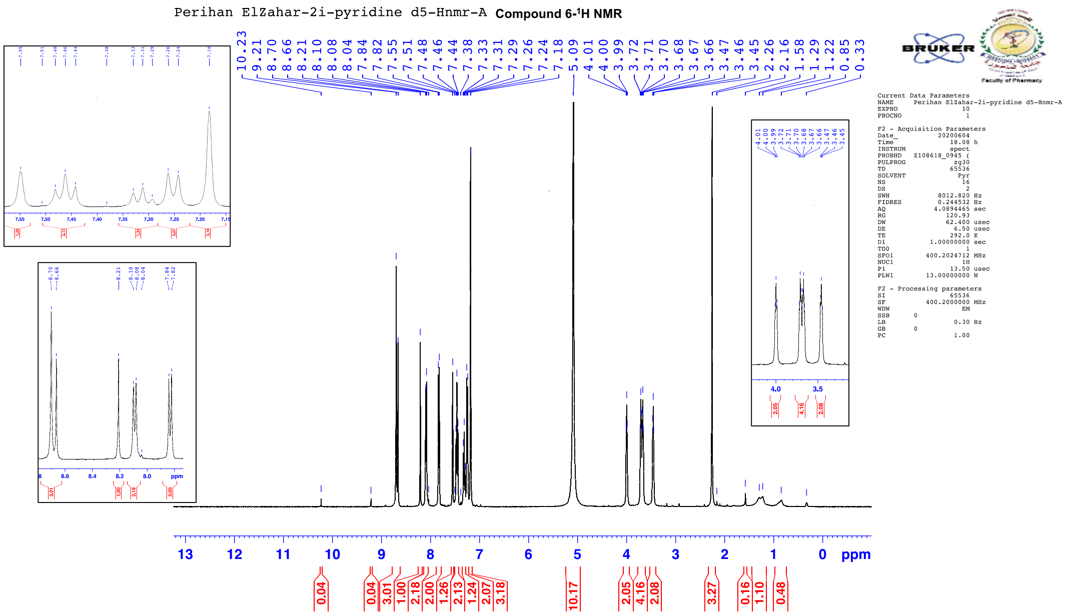

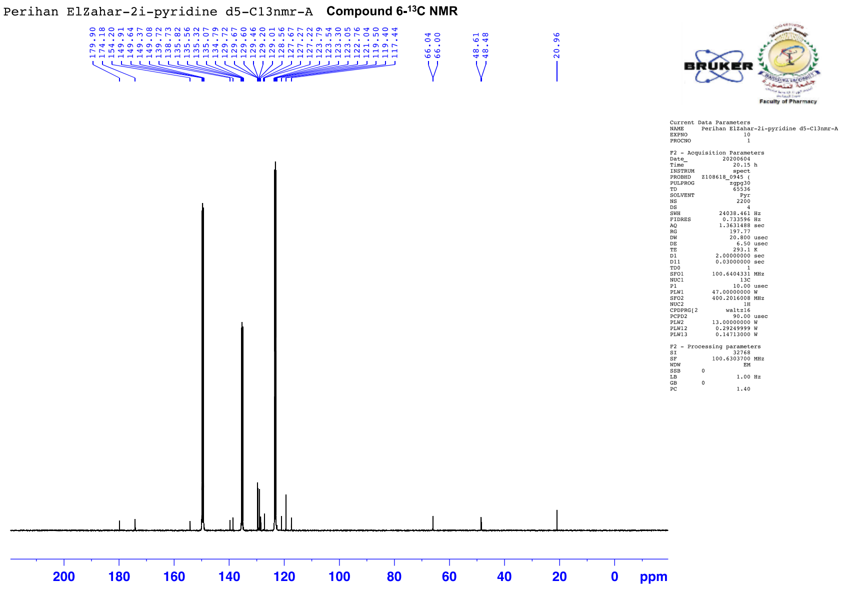

(*Z*)-5-((3-(4-methoxyphenyl)-1-phenyl-1*H*-pyrazol-4-yl)methylene)-2-(piperidin-1-yl)thiazol-4(5*H*)-one **(7)**

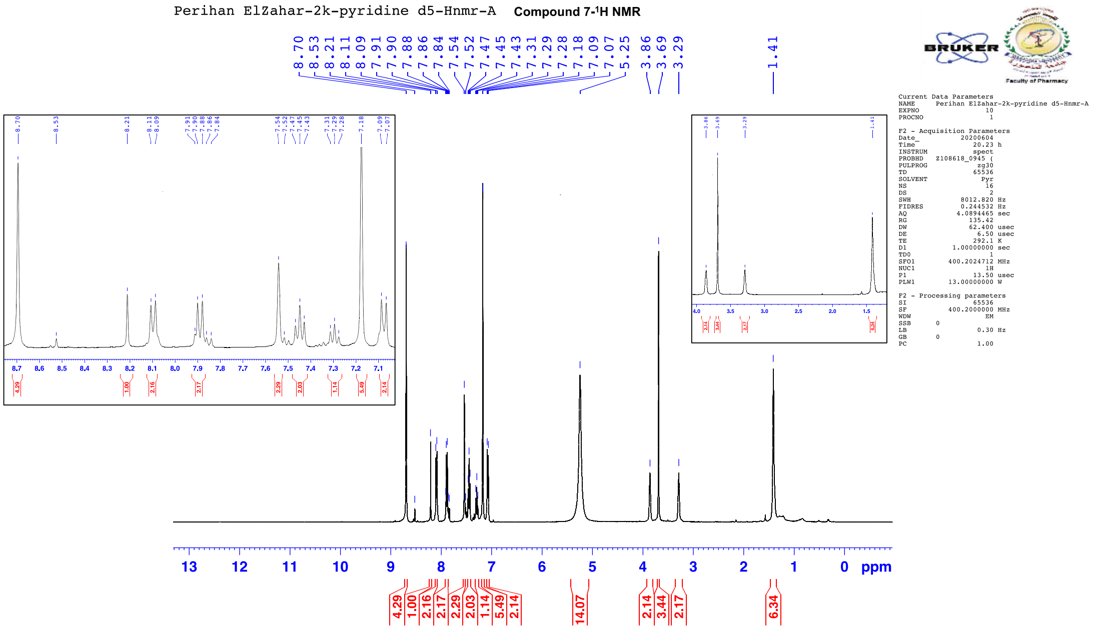

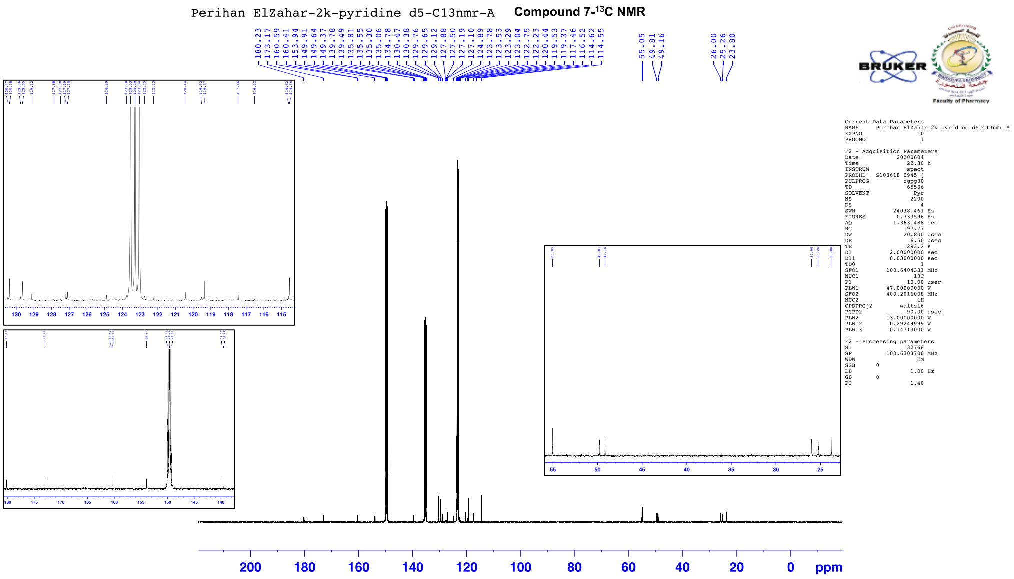

(*Z*)-5-((3-(4-methoxyphenyl)-1-phenyl-1*H*-pyrazol-4-yl)methylene)-2-morpholinothiazol-4(5*H*)-one **(8)**

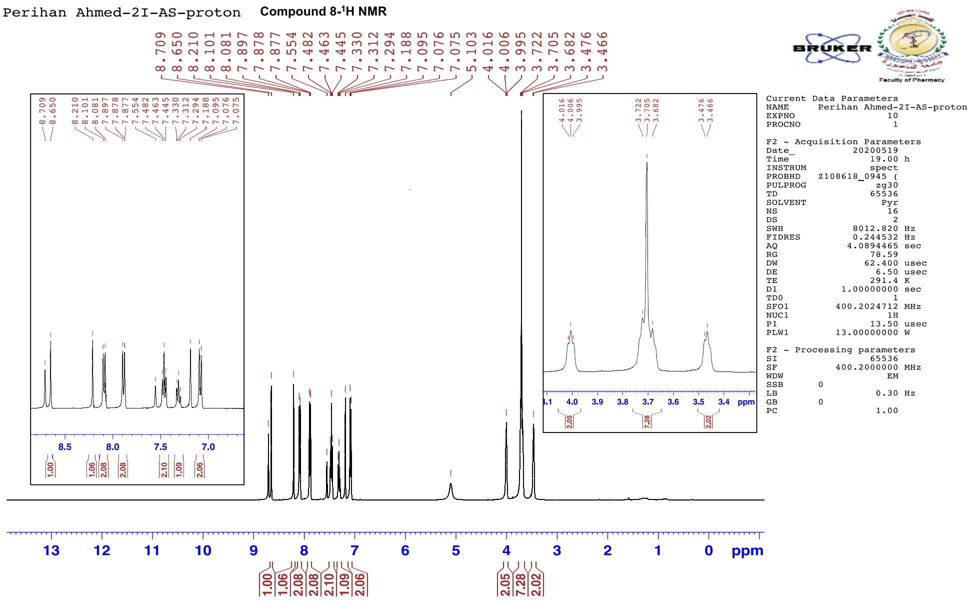

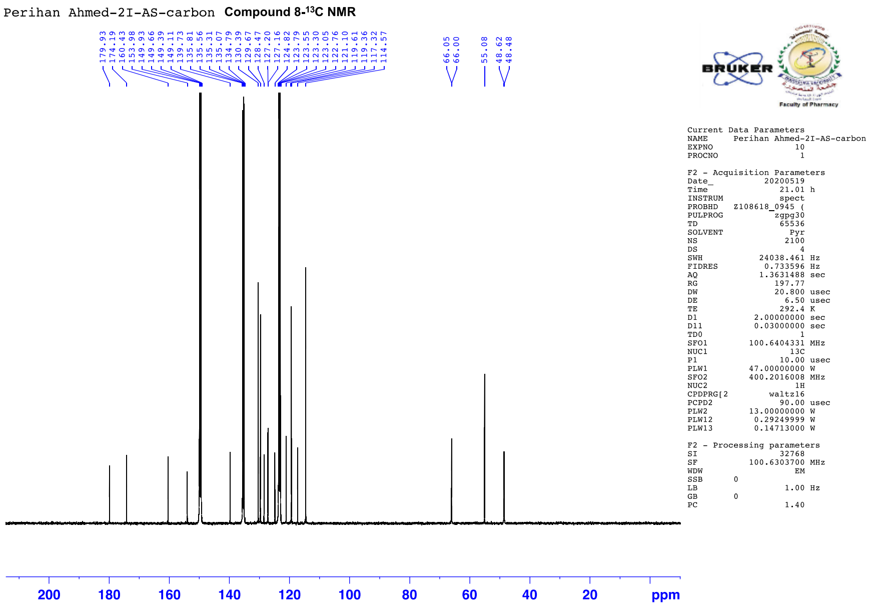

(*Z*)-5-((3-(4-bromophenyl)-1-phenyl-1*H*-pyrazol-4-yl)methylene)-2-(piperidin-1-yl)thiazol-4(5*H*)-one **(9)**

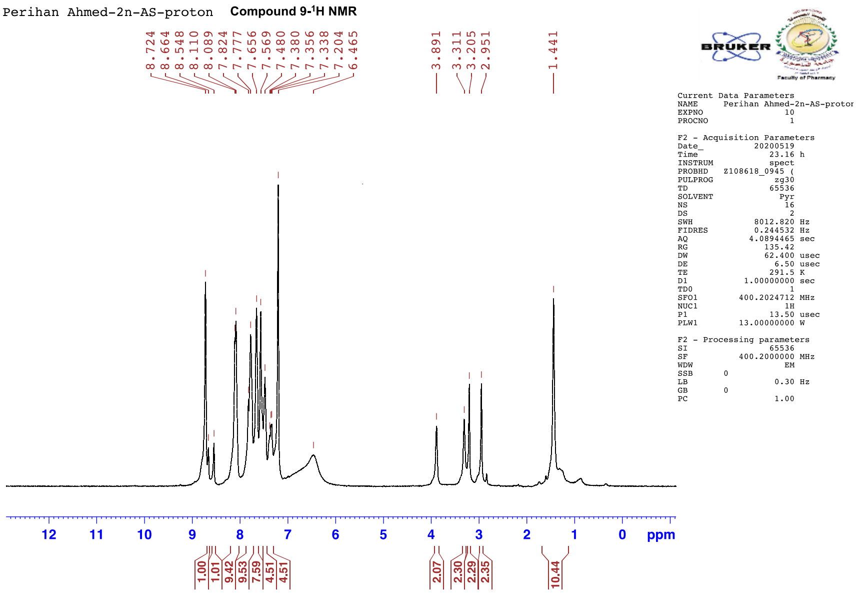

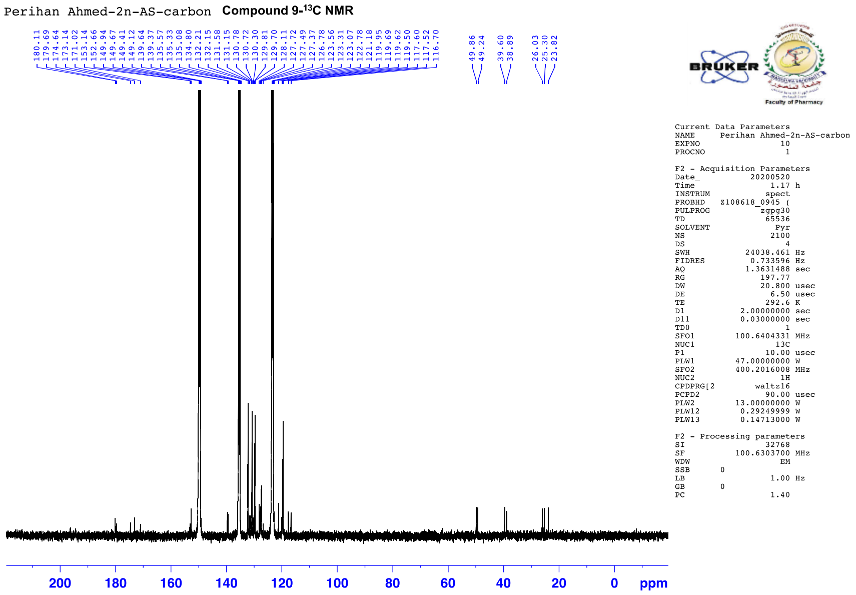

(*Z*)-5-((3-(4-bromophenyl)-1-phenyl-1*H*-pyrazol-4-yl)methylene)-2-morpholinothiazol-4(5*H*)-one **(10)**

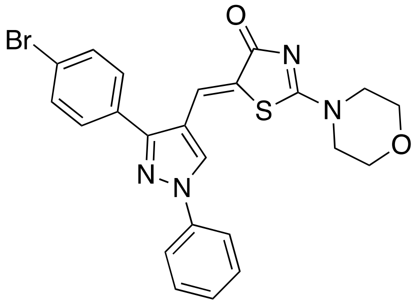

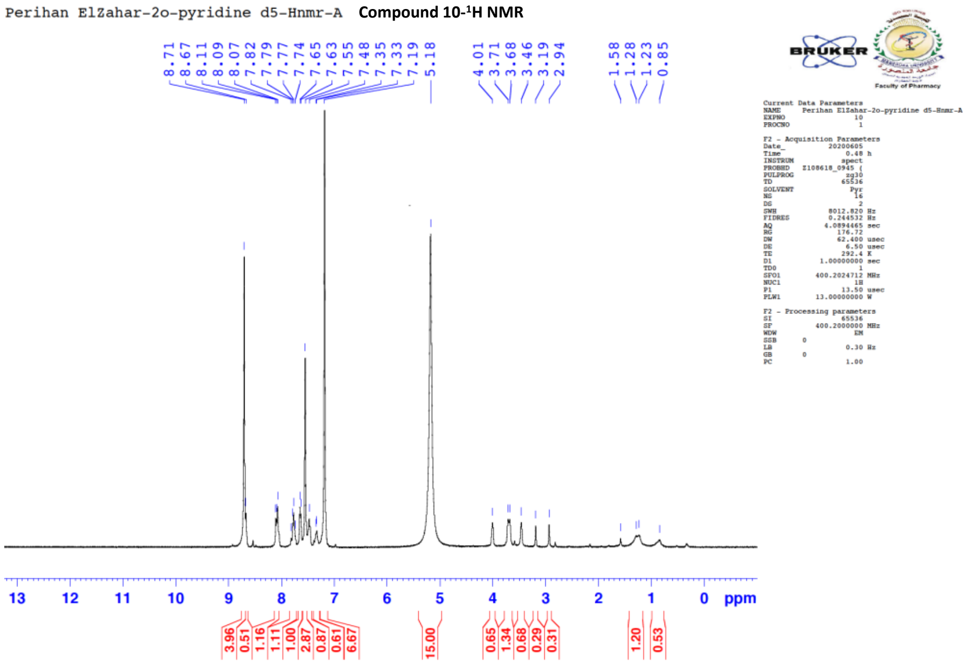

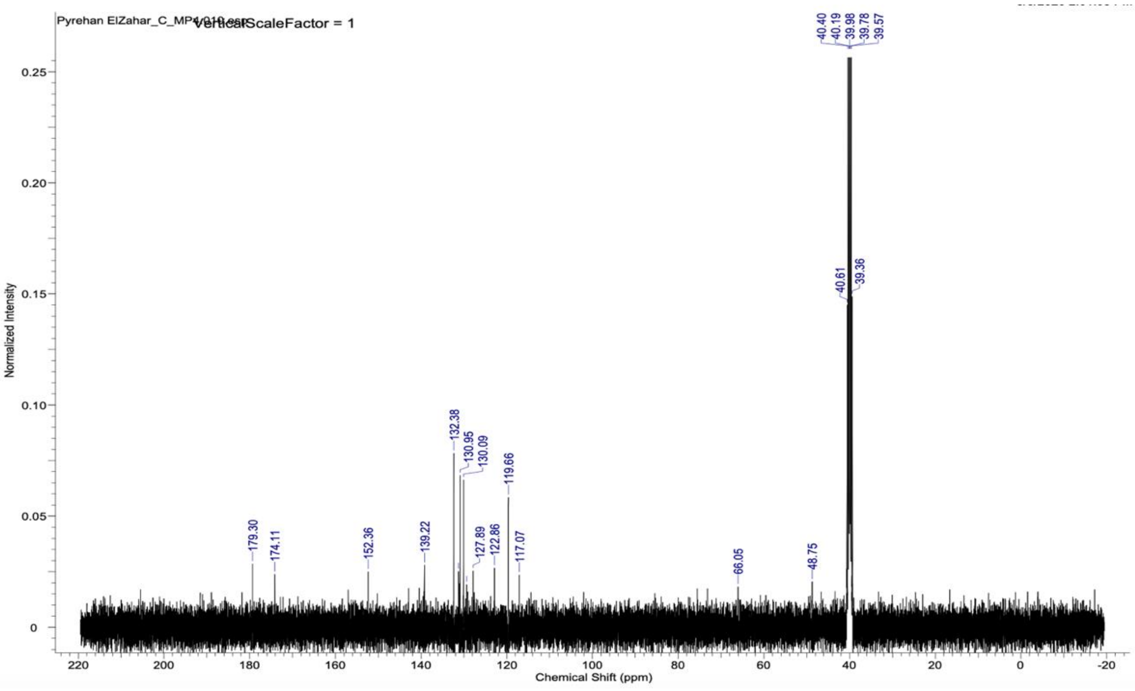

Ascertainment of purity by HPLC:

Compound 1

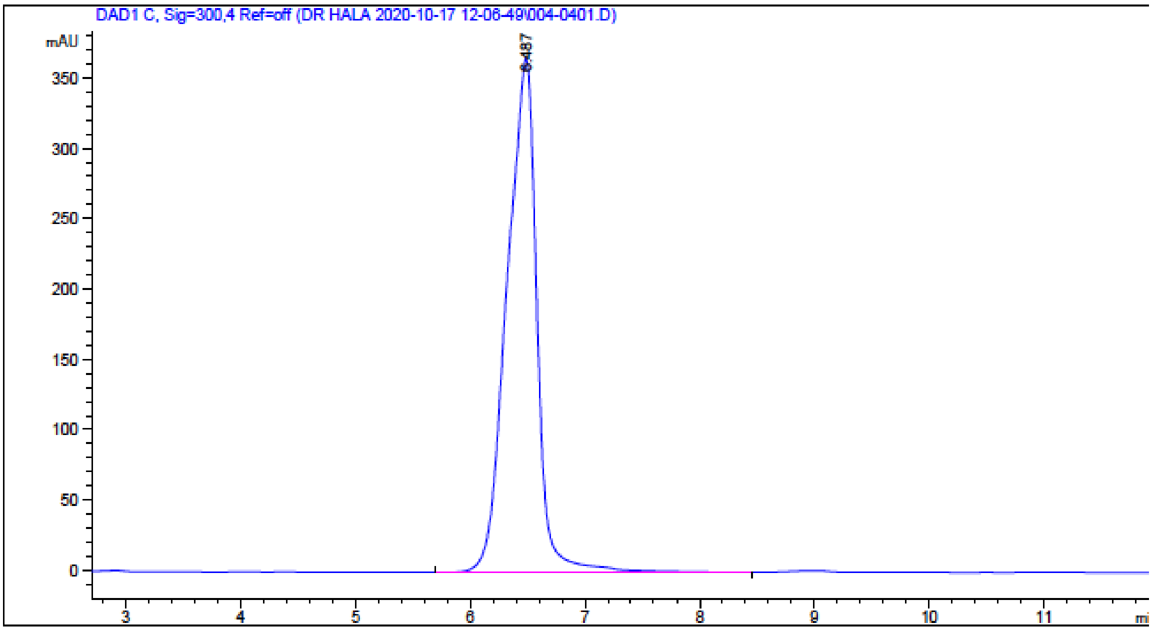


```
=====================================================================
                          Area Percent Report
=====================================================================

Sorted By             :      Signal
Multiplier:                   :      1.0000
Dilution:                     :      1.0000
Use Multiplier & Dilution Factor with ISTDs


Signal 1: DAD1 C, Sig=300,4 Ref=off

Peak RetTime Type  Width     Area      Height     Area
  #    [min]        [min]   [mAU*s]     [mAU]       %
----|-------|----|-------|----------|----------|--------|
   1   6.487 BB     0.2542 6646.30127  365.42575 100.0000

Totals :                   6646.30127  365.42575
```

Compound 2

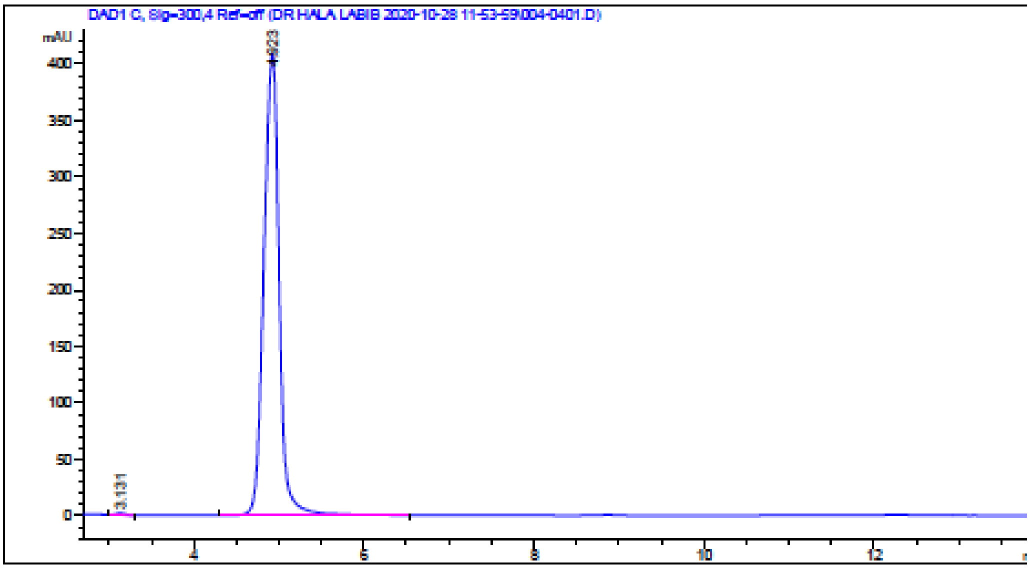


```
=====================================================================
                         Area Percent Report
=====================================================================

Sorted By             :      Signal
Multiplier:                   :      1.0000
Dilution:                     :      1.0000
Use Multiplier & Dilution Factor with ISTDs


Signal 1: DAD1 C, Sig=300,4 Ref=off

Peak RetTime Type  Width     Area      Height     Area
  #   [min]        [min]   [mAU*s]     [mAU]        %
----|-------|----|-------|----------|----------|--------|
   1   3.131 BB    0.0949   10.64844    1.70346   0.2089
   2   4.923 BB    0.1970 5086.16064  409.10104  99.7911

Totals :                  5096.80909  410.80450
```

Compound 3

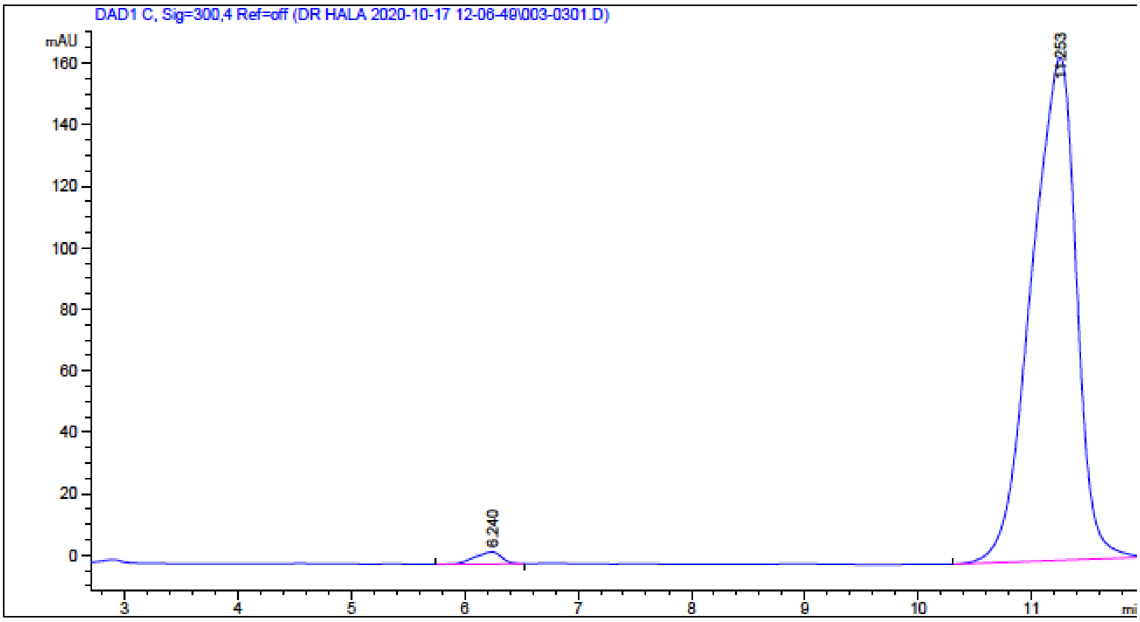

===
Area Percent Report
===

Sorted By : Signal
Multiplier: : 1.0000
Dilution: : 1.0000
Use Multiplier & Dilution Factor with ISTDs

Signal 1: DAD1 C, Sig=300,4 Ref=off

Peak #	RetTime [min]	Type	Width [min]	Area [mAU*s]	Height [mAU]	Area %
1	6.240	BB	0.2313	63.82434	3.93368	1.4020
2	11.253	BBA	0.4501	4488.63086	163.58569	98.5980
Totals :				4552.45520	167.51937	

Compound 4

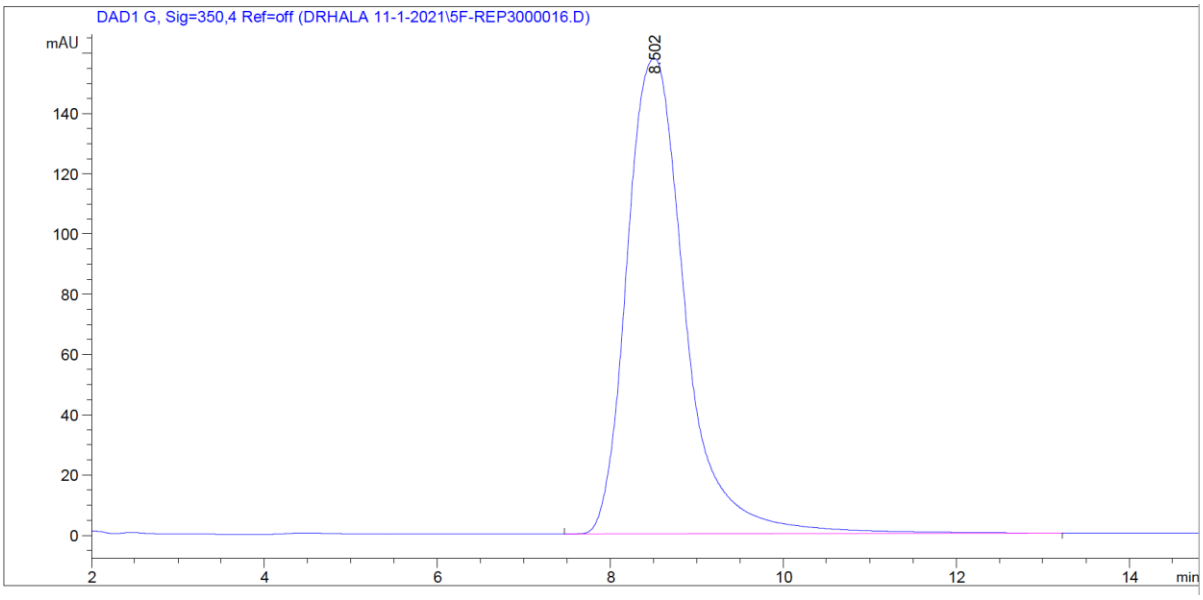


```
=====================================================================
                         Area Percent Report
=====================================================================

Sorted By             :      Signal
Multiplier:                  :       1.0000
Dilution:                    :       1.0000
Use Multiplier & Dilution Factor with ISTDs


Signal 1: DAD1 G, Sig=350,4 Ref=off

Peak RetTime Type  Width     Area      Height     Area
  #   [min]        [min]   [mAU*s]     [mAU]        %
----|-------|----|-------|----------|----------|--------|
   1   8.502 BB    0.7390 7401.62012  157.87465 100.0000

Totals :                  7401.62012  157.87465


=====================================================================
```

Compound 5

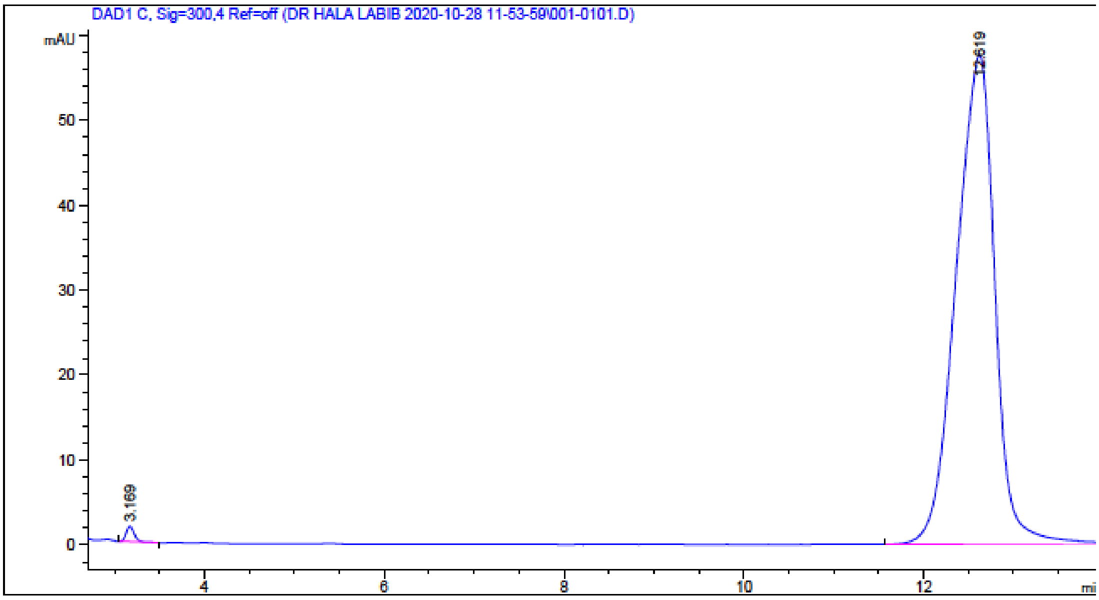


```
=====================================================================
                         Area Percent Report
=====================================================================

Sorted By             :      Signal
Multiplier:                   :      1.0000
Dilution:                     :      1.0000
Use Multiplier & Dilution Factor with ISTDs


Signal 1: DAD1 C, Sig=300,4 Ref=off

Peak RetTime Type  Width     Area      Height     Area
  #   [min]        [min]   [mAU*s]     [mAU]        %
----|-------|----|-------|----------|----------|--------|
   1   3.169 BB    0.0984   11.49168    1.80150   0.6628
   2  12.619 BB    0.4831 1722.22546   57.56257  99.3372

Totals :                  1733.71714   59.36407
```

Compound 6

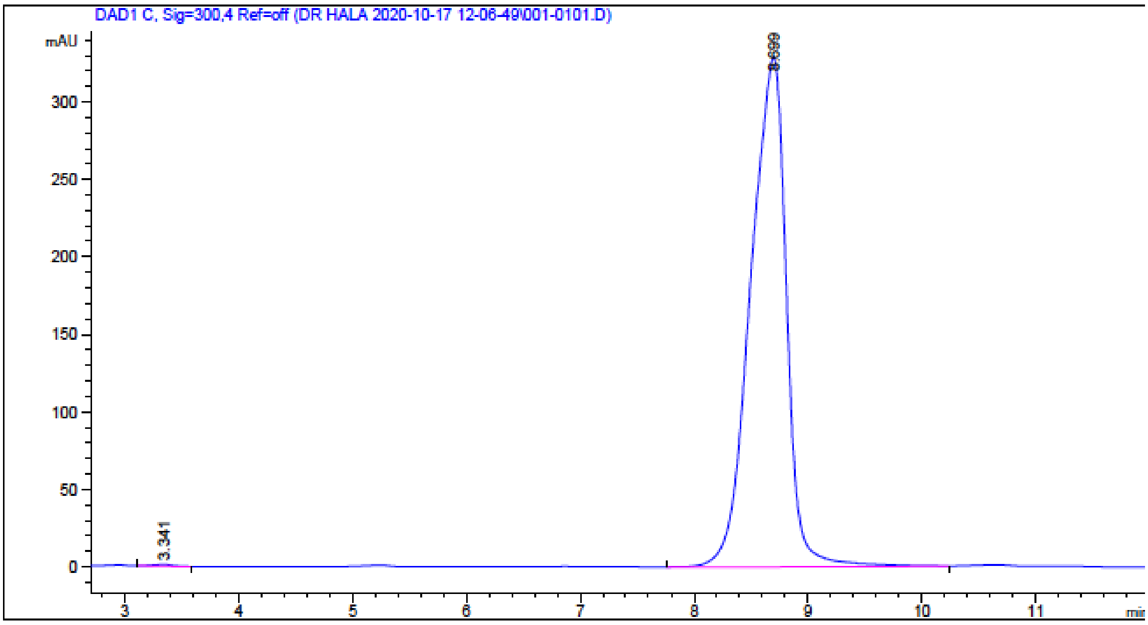

===

Area Percent Report

===

Sorted By : Signal
Multiplier: : 1.0000
Dilution: : 1.0000
Use Multiplier & Dilution Factor with ISTDs

Signal 1: DAD1 C, Sig=300,4 Ref=off

Peak #	RetTime [min]	Type	Width [min]	Area [mAU*s]	Height [mAU]	Area %
1	3.341	BB	0.1495	14.48479	1.34662	0.2009
2	8.699	BB	0.3575	7194.99170	328.52420	99.7991

Totals : 7209.47649 329.87082

Compound 7

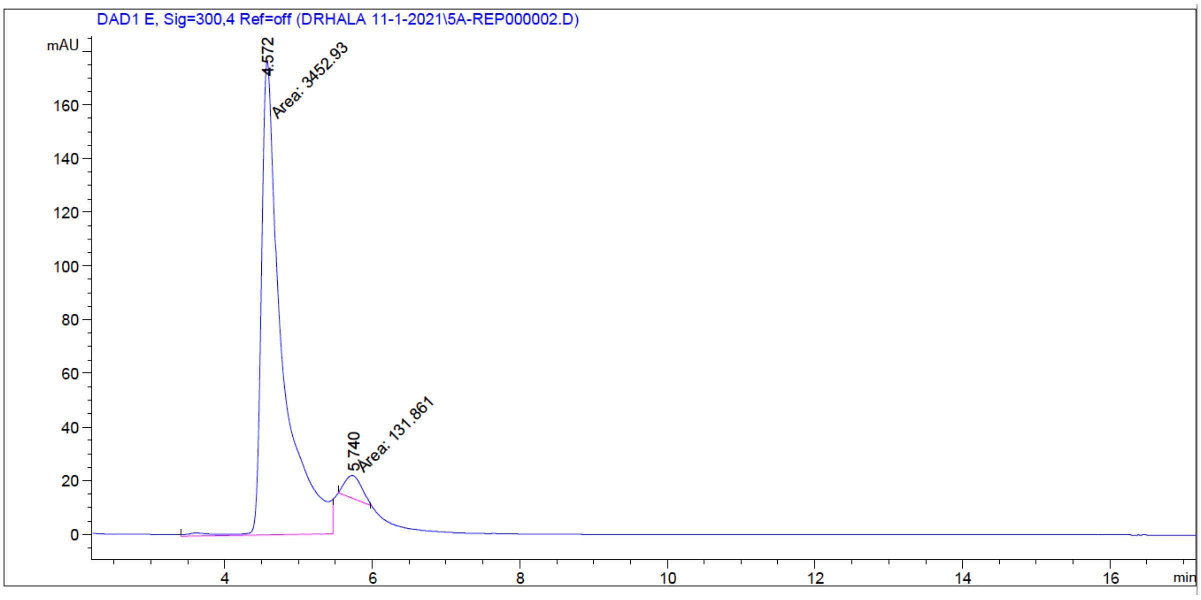

==

Area Percent Report

==

Sorted By : Signal
Multiplier: : 1.0000
Dilution: : 1.0000
Use Multiplier & Dilution Factor with ISTDs

Signal 1: DAD1 E, Sig=300,4 Ref=off

Peak #	RetTime [min]	Type	Width [min]	Area [mAU*s]	Height [mAU]	Area %
1	4.572	MM	0.3254	3452.93140	176.85864	96.3217
2	5.740	MM	0.2593	131.86057	8.47412	3.6783

Totals : 3584.79196 185.33276

==

Compound 8

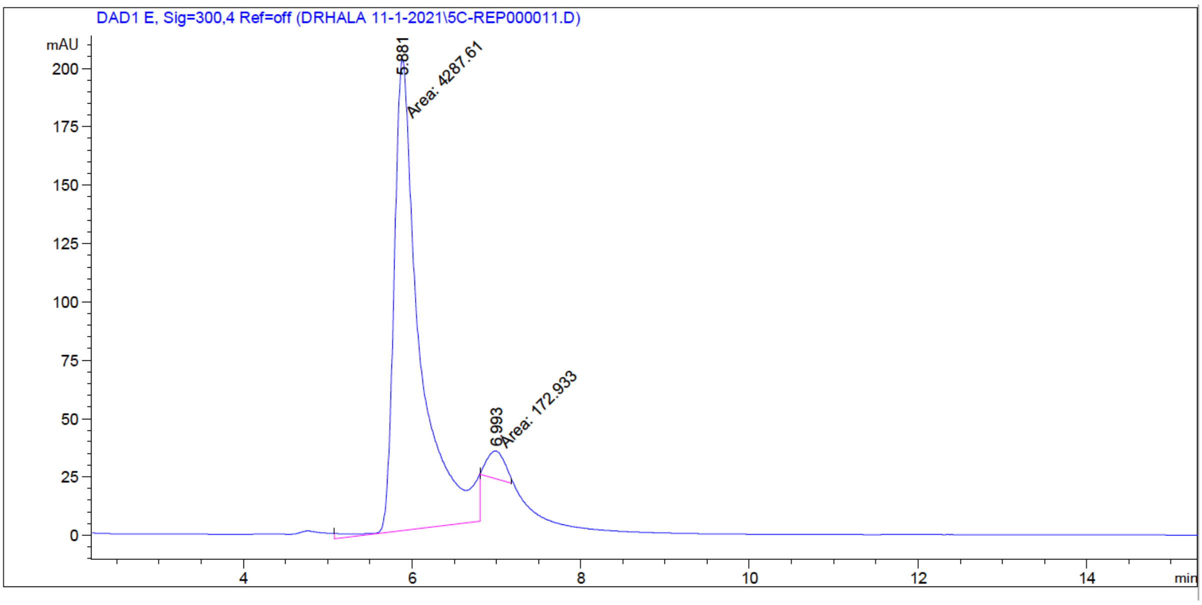

==

Area Percent Report

==

Sorted By : Signal
Multiplier: : 1.0000
Dilution: : 1.0000
Use Multiplier & Dilution Factor with ISTDs

Signal 1: DAD1 E, Sig=300,4 Ref=off

Peak #	RetTime [min]	Type	Width [min]	Area [mAU*s]	Height [mAU]	Area %
1	5.881	MM	0.3538	4287.61377	201.96274	96.1231
2	6.993	MM	0.2424	172.93280	11.88936	3.8769

Totals : 4460.54657 213.85210

==

Compound 9

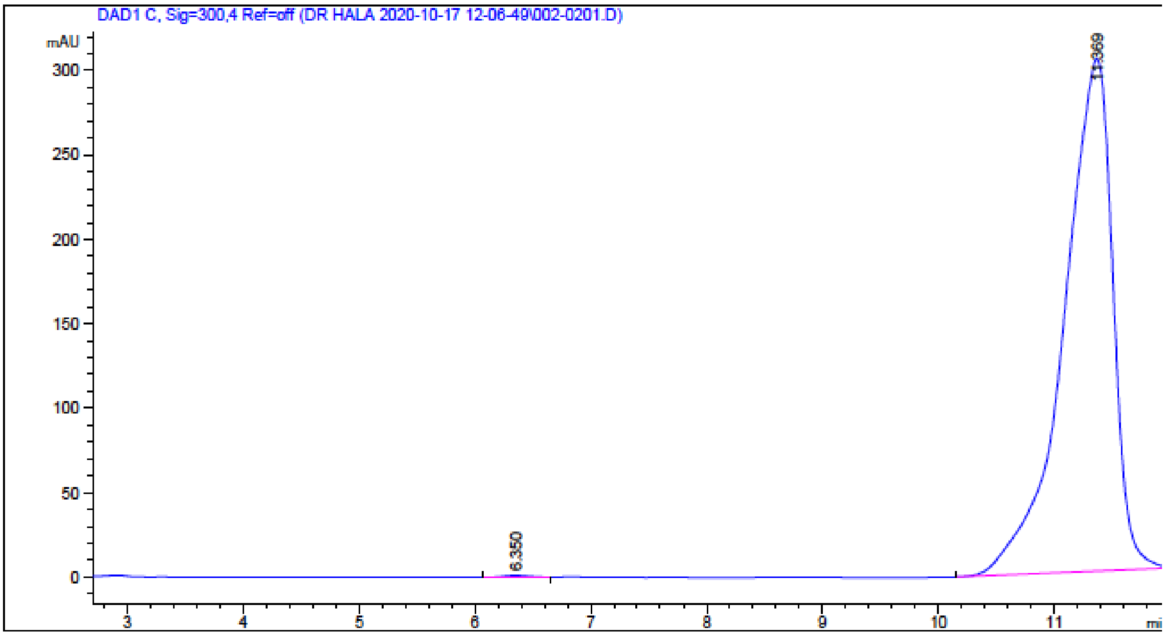

===
Area Percent Report
===

Sorted By : Signal
Multiplier: : 1.0000
Dilution: : 1.0000
Use Multiplier & Dilution Factor with ISTDs

Signal 1: DAD1 C, Sig=300,4 Ref=off

Peak #	RetTime [min]	Type	Width [min]	Area [mAU*s]	Height [mAU]	Area %
1	6.350	BB	0.2304	16.19253	1.07098	0.1818
2	11.369	BBA	0.4641	8892.33887	303.28870	99.8182

Totals : 8908.53140 304.35968

Compound 10

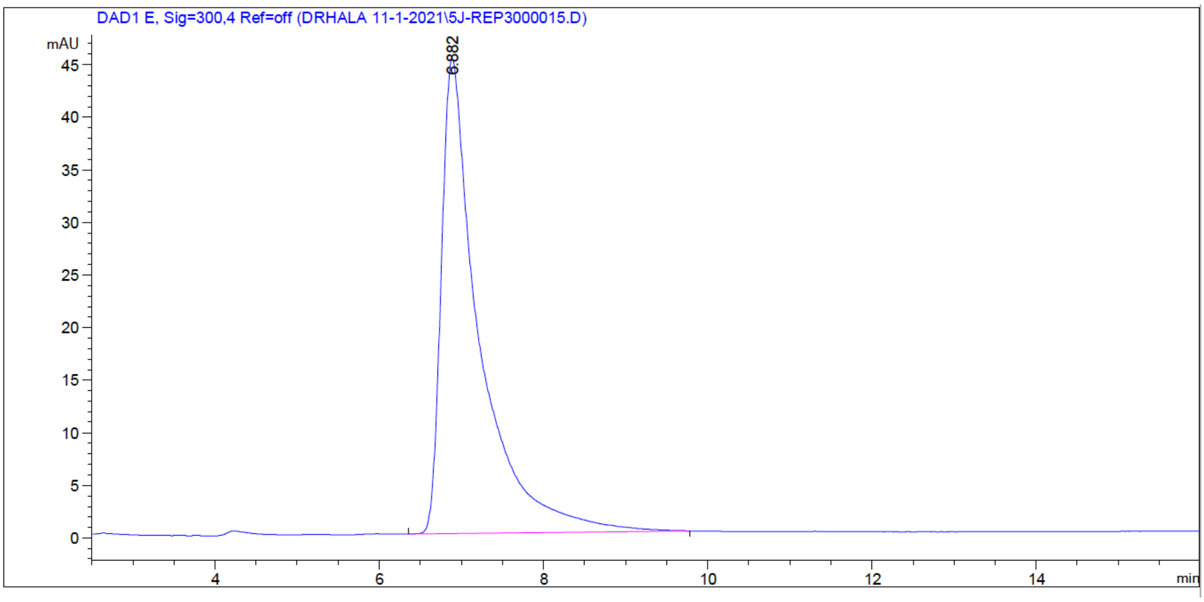


```
=====================================================================
                         Area Percent Report
=====================================================================

Sorted By             :      Signal
Multiplier:                  :       1.0000
Dilution:                    :       1.0000
Use Multiplier & Dilution Factor with ISTDs


Signal 1: DAD1 E, Sig=300,4 Ref=off

Peak RetTime Type  Width      Area       Height     Area
  #    [min]        [min]    [mAU*s]     [mAU]        %
----|-------|----|-------|----------|----------|--------|
   1   6.882 BB    0.4518 1460.27197   45.09696 100.0000

Totals :                  1460.27197   45.09696


=====================================================================
```
